# Genetic evaluation of the evolutionary distinctness of a federally endangered butterfly, Lange’s Metalmark

**DOI:** 10.1186/s12862-015-0354-9

**Published:** 2015-04-25

**Authors:** Benjamin Proshek, Julian R Dupuis, Anna Engberg, Ken Davenport, Paul A Opler, Jerry A Powell, Felix AH Sperling

**Affiliations:** Department of Biological Sciences, University of Alberta, Edmonton, AB Canada; Essig Museum of Entomology, University of California, Berkeley, CA USA; C.P. Gillette Museum of Arthropod Diversity, Colorado State University, Ft. Collins, CO USA

**Keywords:** Conservation, Mitochondrial DNA, Population genetics, Phylogeny, Evolutionarily significant unit, ESU, *Apodemia mormo langei*, Riodinidae, Endangered species act, Conservation prioritization

## Abstract

**Background:**

The Mormon Metalmark (*Apodemia mormo*) species complex occurs as isolated and phenotypically variable colonies in dryland areas across western North America. Lange’s Metalmark, *A. m. langei*, one of the 17 subspecies taxonomically recognized in the complex, is federally listed under the U.S. Endangered Species Act of 1973. Metalmark taxa have traditionally been described based on phenotypic and ecological characteristics, and it is unknown how well this nomenclature reflects their genetic and evolutionary distinctiveness. Genetic variation in six microsatellite loci and mitochondrial *cytochrome oxidase subunit I* sequence was used to assess the population structure of the *A. mormo* species complex across 69 localities, and to evaluate *A. m. langei*’s qualifications as an Evolutionarily Significant Unit.

**Results:**

We discovered substantial genetic divergence within the species complex, especially across the Continental Divide, with population genetic structure corresponding more closely with geographic proximity and local isolation than with taxonomic divisions originally based on wing color and pattern characters. Lange’s Metalmark was as genetically divergent as several other locally isolated populations in California, and even the unique phenotype that warranted subspecific and conservation status is reminiscent of the morphological variation found in some other populations.

**Conclusions:**

This study is the first genetic treatment of the *A. mormo* complex across western North America and potentially provides a foundation for reassessing the taxonomy of the group. Furthermore, these results illustrate the utility of molecular markers to aid in demarcation of biological units below the species level. From a conservation point of view, *Apodemia mormo langei*’s diagnostic taxonomic characteristics may, by themselves, not support its evolutionary significance, which has implications for its formal listing as an Endangered Species.

**Electronic supplementary material:**

The online version of this article (doi:10.1186/s12862-015-0354-9) contains supplementary material, which is available to authorized users.

## Background

Global loss of biodiversity, fueled by unprecedented anthropogenic influences, has elevated the importance of conservation biology in mainstream public consciousness. Despite this increased attention, one of the most fundamental challenges of conservation remains unsolved and contentious: how do we accurately identify and delimit manageable units of biodiversity [1-9]? Since species are one of the fundamental units of biology, this challenge is rooted in taxonomy [10-13]. Successful conservation, however, also relies on our understanding of diversity below the species level [14], as evolutionary and ecological potential is often recognized at the population level [6,15].

Evolutionarily (or Evolutionary) Significant Units (ESUs) were originally developed to facilitate objective prioritization of conservation units (CUs) below the species level [16]. At a time when conservation managers (particularly in mammalian systems) were grappling with sometimes-trivial subspecific classifications [16], ESUs aimed to refocus conservation resources on populations exhibiting the most distinct evolutionary characteristics. However, as with species concepts in taxonomy (e.g. [17]), alternate definitions and operational criteria of ESUs have proliferated [1,3-8,18-20], as have alternate qualifiers for CUs (e.g. management units (MUs)/demographic units (DUs) [2,14], discrete population segments (DPSs) [21], and service-providing units (SPUs) [14]). Starting with relatively conceptual, integrative origins [1,16] that were criticized for their subjectivity [22], various redefinitions of the ESU have focused on more objective methods for discriminating populations that have evolutionary potential, such as: 1) the use of consistently congruent gene phylogenies [19], 2) reciprocal monophyly of mitochondrial DNA (mtDNA) or allele frequency divergence at nuclear loci [2], and 3) diagnostic characters (including ecological, behavioral, *etc.*) that exclusively cluster individuals or populations using the phylogenetic species concept [4]. While alleviating some subjectivity, these redefinitions met criticism focused on inconsistent phylogenetic reconstruction [23], the stringency of reciprocal monophyly and diagnostic characters [6,7], and the utility of the phylogenetic species concept [3].

In the midst of this operational debate, Crandall *et al.* [6] have argued that a dichotomous designation (“ESU or not”) betrays the goal of ESUs and undermines biological complexity. They presented eight categories to discern population distinctiveness based on genetic and ecological exchangeability *sensu* [24], and proposed that dichotomous use of the term ESU be abandoned in favor of a more holistic approach [6]. Along similar lines, Fraser & Bernatchez [7] contended that all ESU concepts share the same “fundamental essence” and goal, but differ in specific optimality criteria. Following the unified species concept [25], these authors described a framework for adaptive evolutionary conservation that recognizes the situational strengths and weaknesses of each ESU definition and integrates them to define biologically meaningful ESUs anywhere along the evolutionary continuum [7]. In the past 15 years, conceptual redefinitions of the ESU have been replaced by new alternate classifications and subdivisions of CUs. de Guia & Saitoh [8] proposed the use of full and partial ESUs to distinguish populations described based on knowledge of both neutral and adaptive genetic variation from those described using only one aspect of their variation, respectively. Demographic or management units (DUs or MUs, referred to as DUs from this point on) that describe demographically independent populations (generally within ESUs), have also become commonplace alongside the more commonly discussed ESU [14,15,26].

Balancing the complexity of the evolutionary continuum with the practical inventory needs of conservation management is an Augean task, and it can be instructive to reevaluate old decisions. Here, we reassess the conservation status of an endangered butterfly in California in light of its range-wide population structure and phylogeny. Lange’s Metalmark butterfly, *Apodemia mormo langei* [43], is an endangered subspecies characterized by a constrained range and a unique phenotype. Described from the banks of the Sacramento-San Joaquin River system downstream of Sacramento, California, this single site remains the only recognized location for this taxon. Due to habitat loss from sand mining and other activities, and these butterflies’ low vagility and constrained distribution, the population is extremely geographically restricted, and was placed on the US Endangered Species List in 1976 under the Endangered Species Act (ESA) (Federal Register 41:22044, 1976). The Antioch Dunes National Wildlife Refuge (NWR) was established in 1980 to protect *A. m. langei* as well as two species of wildflowers (the Antioch Dunes evening-primrose, *Oenothera deltoides* subsp. *howellii* [Munz] W. Klein and the Contra Costa wallflower*, Erysimum capitatum* var. *angustatum* [Greene] G. Rossb.). Lange’s Metalmark has undergone several boom-and-bust cycles, but is being maintained through extensive conservation efforts by several organizations, including a captive rearing program [27-31].

*Apodemia mormo langei* is a member of a variable species group, the *A. mormo* species complex. Three species are currently recognized in this complex: *A. mormo* [32], *A. virgulti* [33], and *A. mejicanus* [33,34]. *Apodemia mormo* occurs across western North America, from Mexico to Canada, and is by far the widest ranging metalmark species (Riodinidae) in North America. *Apodemia virgulti* and *A. mejicanus* are found in the American Southwest and western Mexico [35], and *A. mejicanus* also has an isolated population in Colorado [36]. The species complex shows considerable variation in wing markings, voltinism, flight periods, host use, and oviposition behavior [37]. Although most feed exclusively on plants in the genus *Eriogonum* (wild buckwheat, Polygonaceae), some also feed on *Krameria* (Rhatany, Krameriaceae) [38]. Due to this variability, most of which is found in the Southwest region of the USA, there is significant taxonomic interest in the group. Currently 17 described subspecies are recognized in the complex [34], but the number and status of these taxa is far from settled (e.g. [39-41]).

In this study, we investigate the identification and demarcation of biological units in the *A. mormo* species complex, from both an evolutionary biology and a conservation biology point of view. We use mitochondrial gene sequence data and microsatellite markers to describe range-wide population structure and phylogenetic relationships in the *A. mormo* species complex, including the endangered subspecies *A. m. langei*. To objectively relate our genetic assessment of the group to preexisting taxonomy, we compare a selection of morphological characters based on taxonomic designations to the genetic diversity across the species complex. In light of our new genetic data, we then reassess the conservation status of *A. m. langei* under various definitions of CUs.

## Results

### mtDNA

**Figure 1:**
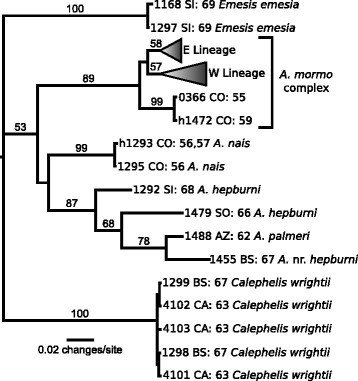
Maximum-likelihood tree of all unique COI haplotypes outside the *A. mormo* group: Letters indicate state or province where collected: AZ: Arizona; BS: Baja Sur; CA: California; CO: Colorado; SI: Sinaloa; SO: Sonora. Locality numbers are given after state/province codes. Numbers above branches indicate bootstrap support.

**Figure 2:**
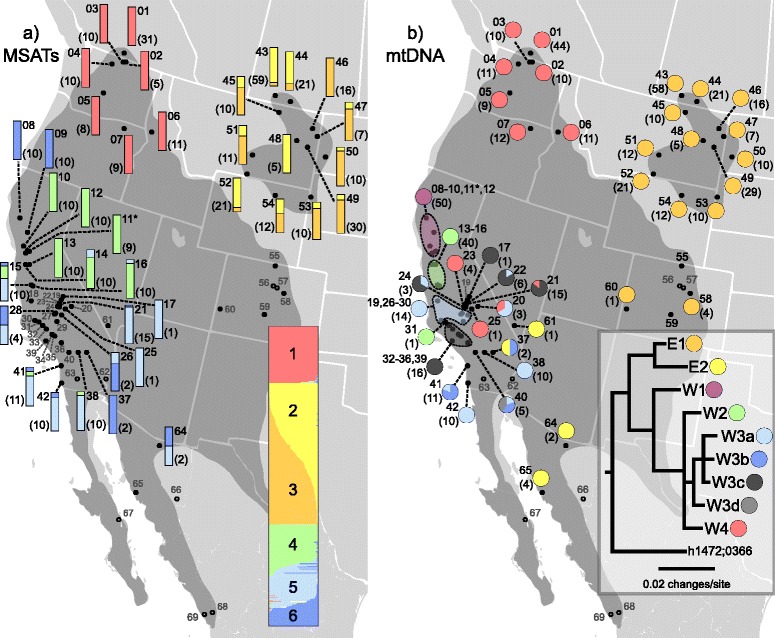
Microsatellite and mitochondrial DNA geographic patterns: population level microsatellite clustering **(a)** and membership in main mtDNA clades **(b)**. Colored bars and pie charts indicate population membership in six STRUCTURE clusters **(a)**, or nine main mtDNA clades **(b)**, respectively. Shaded area indicates the approximate range of the *Apodemia mormo* species complex, modified from [95,38,96-98]. Solid dots represent locations where specimens of the *A. mormo* species complex were collected, and hollow dots indicate locations where outgroups were collected. Non-parenthetical numbers correspond to location numbers (additional file 1: Table S1) and parenthetical numbers indicate the number of specimens per site per dataset. In the case of mtDNA, several groups of populations belonging to the same clade were combined, and sample size for these groups correspond to those populations combined. Grey, non-parenthetical numbers indicate locations for which data was not collected for that dataset. Overall STRUCTURE plot and a simplified tree from additional file 1: Figure S1 are shown in insets. Numbers in the overall STRUCTURE plot correspond to groupings in additional file 1: Figure S1. Divergent *A. mormo* haplotypes h1472 and 0366 (Figure 1) were found in locality numbers 55 and 59. The population of *A. mormo langei* at Antioch Dunes (locality 11) is marked with an asterisk.

We sequenced the entire *cytochrome oxidase subunit I* (COI) gene for 469 specimens and conducted a maximum-likelihood search of 205 unique haplotypes (Figure 1). All specimens of the *A. mormo* complex fell into two clades denoted as the Western and the Eastern lineage, except for the three specimens from the eastern slope of Colorado (localities 55 and 59, Figure 2) which were 3.35% divergent from other individuals in adjacent localities. The Eastern lineage comprised all *A. mormo* complex haplotypes from individuals on or east of the Continental Divide, as well as from locality 60 (Figure 2) on the western slope of the Colorado Rockies. It also included two Sonora, MEX sites (localities 64 & 65), the lone Nevada site (locality 61), and one individual from a site in San Bernardino Co., California (locality 37). The Western lineage comprised all other haplotypes from west of the Continental Divide. The average percent sequence divergence between these two clades was 3.07%.

Within the eastern lineage, two major clades are observed: One consisting of the Mexican haplotypes, the one sample from Nevada, and the outlier from San Bernardino, CA (clade E2: Figure 2); and the other containing the remaining eastern haplotypes (clade E1). With the exception of one small clade of haplotypes from Wyoming and Montana with 94% bootstrap support and two distinct monophyletic clades comprised of Montana (localities 51 and 52) and Saskatchewan & Montana haplotypes (localities 43, 44, and 45), all other clades showed shallow relationships with little geographic pattern (see Additional file 1: Figure S1 for complete trees).

The Western lineage was composed of four major clades, labeled W1-W4 (Figure 2, Additional file 1: Figure S1). Clade W1 was composed of specimens from the five northernmost collection locations in California (localities 8–12), including the Antioch Dunes population of *A. m. langei*. Although branch lengths within this clade were short, divergence of 2.51% was observed relative to the rest of the western lineage. Relationships between the three remaining western clades were unresolved, but each was moderately divergent from the others. Excluding one outlier from Santa Barbara Co., CA (haplotype 1189, locality 31), clade W2 included specimens from four geographically intermediate populations in central California (localities 13–16) as well as one in south central California (locality 31). Clade W3 was comprised of the majority of the individuals from south-central and southern California. Finally, clade W4 included all individuals from the Pacific Northwest, but interestingly, also included several individuals from south-central Californian populations (localities 20, 21, 23, 25). The only western lineage to exhibit significant substructure was Clade W3, which displayed four main internal clades (W3a-W3d). Divergence of these clades ranged from 0.94% to 1.10%, but exhibited little geographic pattern.

Regionally, tests of neutrality (Tajima’s *D* and Fu’s *F*_*s*_) agreed and rejected the null hypothesis of constant population size only for populations east of the Continental Divide (Table 1). All other Tajima’s *D* statistics were not statistically significant. Fu’s *F*_*s*_ statistics was significant globally and for California regionally, and supported models of population growth or purifying selection in both cases. No statistically significant signatures of population bottlenecks/overdominant selection were observed regionally, or in any populations individually (Table 1; California: Additional file 1: Table S4; Pacific Northwest and East of the Continental Divide: [42]).

**Table 1:** **Descriptive statistics and tests of isolation by distance: Descriptive statistics, population differentiation values, and isolation by distance (IBD) results for all microsatellite data (Global) and for localities separated regionally (PNW: Pacific Northwest; East: east of the Continental Divide; CA: California)**

**Region**	**avg. H**_**obs**_ **(95% CI)**	**Alleles/locus**	**F**_**ST**_	**IBD** ***r*** ^***2***^	**Tajima’s** ***D***	**Fu’s** ***F***_***s***_
Global	0.5072 (0.4586-0.5558)	4.93	0.2496	**0.06558**	0.18606	**−23.42845**
PNW	0.2504 (0.2169-0.2839)	2.32	0.1190	**0.19293**	2.50161	9.54345
East	0.6198 (0.5895-0.6501)	6.00	0.0685	**0.11585**	**−1.60431**	**−25.40856**
CA	0.5571 (0.5043-0.6099)	5.52	0.2047	0.00537	−0.06426	**−12.38967**

Alleles/locus refers to the average number of alleles per locus. Bolded numbers indicate significant values (*p* < 0.05).

### Microsatellites

Six microsatellite loci were genotyped for 447 specimens. Across all localities, observed heterozygosity ranged from 0.1905 (locality 7) to 0.7578 (locality 10), and strong population differentiation was observed overall (F_ST_ = 0.2496: Table 1). In pair-wise tests of differentiation, most population pairs were significantly differentiated (only 13 out of 595 pair-wise comparisons were not significantly differentiated). When considering sets of populations regionally (Pacific Northwest, east of the divide, and California), the Pacific Northwest showed statistically lower heterozygosity (pair-wise tests: PNW vs. CA: W = 176, *p* = <0.00001; PNW vs. East: W = 0, *p* = <0.00001; CA vs. East: W = 86, *p* = 0.14163), while populations east of the Continental divide showed the lowest genetic differentiation. Considering all localities, there was a weak, but significant signature of IBD (Table 1). Regionally, significant signatures were observed in the Pacific Northwest and east of the Continental Divide, but not in California. Observed heterozygosity and pair-wise F_ST_ values for all populations are provided in Additional file 1: Table S5.

**Figure 3:**
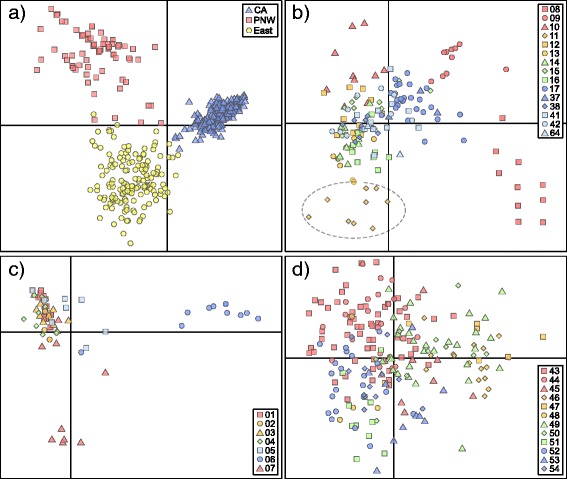
Discriminant analysis of principal components: DAPC based on microsatellite data for **a)** global dataset, and for populations from **b)** California (CA), **c)** the Pacific Northwest (PNW), and **d)** east of the Continental Divide (East). Symbol shapes and colors within each region distinguish localities (locality numbers follow Additional file 1: Table S1), and *A. m. langei* is circled.

In a total analysis of all samples, STRUCTURE estimated the presence of two genetic clusters (K = 2), one corresponding to populations in California and the other corresponding to those in the Pacific Northwest and east of the Continental Divide. Further substructuring divided these two clusters into six (K = 6, Figure 2), and we will focus on these six clusters. Analyzing the populations from west and east of the Continental Divide separately, which enabled removal of the locus that did not amplify for each (see methods: locus E7 west of the divide, locus M2 east of it), resulted in similar clustering to K = 6 (results not shown). Considering K = 6, two clusters corresponded to populations east of the divide (Montana and Saskatchewan), one to the Pacific Northwest, and three to California and Mexico. DAPC estimated a larger number of clusters globally (13–16), but these clusters simply split the three regional groupings (Pacific Northwest, east of the Continental Divide, and California) into several smaller, overlapping clusters (Figure 3a). Overall, DAPC and STRUCTURE both delimited the three main regions.

DAPC and STRUCTURE were also run on regional datasets in an attempt to elucidate population structure at that scale. Regional STRUCTURE analyses did not provide any additional resolution, and all regions mirrored the global analysis (K = 1, 3, and 2 for the Pacific Northwest, California, and east of the Continental Divide; results not shown). DAPC, however, provided resolution for the Pacific Northwest and Californian populations, but not for those east of the Continental Divide (Figure 3b-d). In the Pacific Northwest, the two most geographically distant populations (localities 06 and 07) were separated from a main cluster including the remaining populations (Figure 3c). In California, DAPC clustering roughly matched ancestry estimates from STRUCTURE, although as in the global DAPC analysis, more genetic clusters were estimated (K = 5), which graphically resembled over-splitting of the main groups. Similar to the clustering in the Pacific Northwest, in California, a main cluster was observed that showed some geographic substructure (i.e. geographically proximate populations tend to cluster more closely with each other). Several populations from northern California (localities 08, 09, 10, and 11) were separated from this main cluster, however, including the population of *A. m. langei* at Antioch Dunes (Figure 3b).

**Figure 4:**
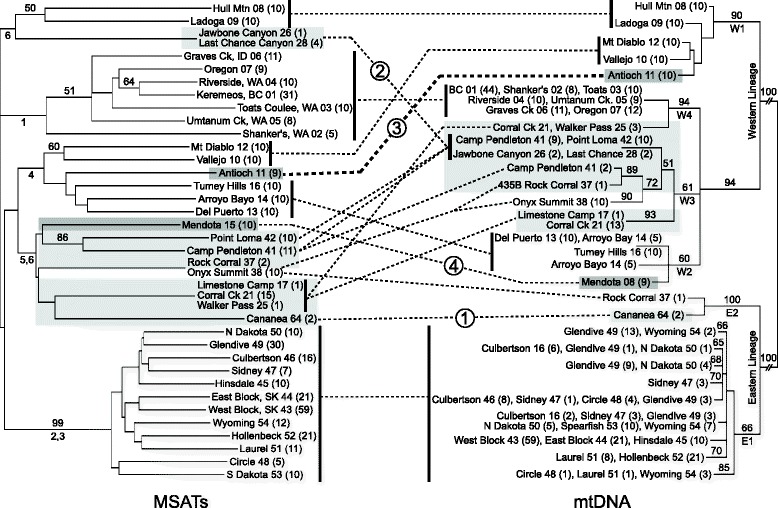
Trees comparing microsatellite and mitochondrial DNA sequence data: Left: Neighbor-joining tree constructed from microsatellite genetic distances, and Right: Maximum-likelihood tree generated from reduced mtDNA dataset. Terminal tips have been manually condensed to simplify groupings in order to show correspondence between trees, indicated by dashed lines; numbered circles on dashed lines correspond to major differences between the trees as mentioned in RESULTS. The thick dashed line indicates *A. m. langei*. Locality names are followed by locality numbers, with sample sizes in parentheses. Shading around branches indicates specimens of *A. m. langei* or *A. m.* nr. *langei* (dark shading), or localities including specimens assigned to a taxon other than *A. mormo* (light shading: *A. virgulti* or *A. mejicanus*). Unshaded branches indicate specimens of *A. mormo*. Numbers above branches indicate bootstrap support, and numbers/letters below branches indicate main mtDNA clades (right) or main STRUCTURE groupings (left: Note that main STRUCTURE groupings are based on population averages, and are not 100% inclusive).

The STRUCTURE clusters corresponded well to the clades found with mtDNA: the two clusters east of the divide corresponded to the Eastern lineage, the Pacific Northwest cluster to Clade W4 of the Western lineage, and the remaining three California/Mexico clusters to clades W1-W3 of the Western lineage. Correspondence between these latter clusters and haplotypes, however, was less straightforward. Figure 4 compares a distance-based tree generated from microsatellite allele frequencies, to a tree generated from a reduced, but comparable, mtDNA dataset (154 haplotypes from samples for which we had microsatellite data). Respectively, these two trees correspond to the clusters from STRUCTURE (for the microsatellite data), and to the overall mtDNA tree (including all haplotypes). When comparing these two trees, several main differences are observed and are numbered as follows in Figure 4: 1) the Cananea, MEX population clusters with the western instead of the eastern samples; 2) the Jawbone and Limestone Camp populations of south-central California cluster with the northern California Hull Mt. and Ladoga samples; 3) the Antioch *Apodemia mormo langei* samples cluster with Tumey Hills, Arroyo Bayo and Del Puerto populations rather than with Mt. Diablo and Vallejo samples; and 4) the Mendota area population clusters with the southern California Camp Pendleton and Point Loma samples rather than with the geographically closer Tumey Hills, Arroyo Bayo and Del Puerto samples.

### Morphology-based taxonomic assignment and concordance to genetic data

**Figure 5:**
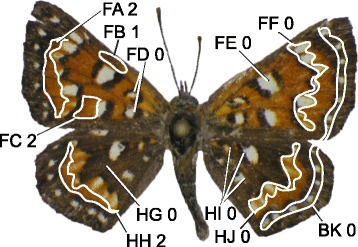
Illustration of eleven wing characters and selected states: In character names, “F” refers to a forewing character, “H” to a hindwing character, and “B” to a character on both pairs of wings. In the descriptions, “DF” refers to “dorsal forewing” and “DH” to “dorsal hindwing”.

In order to objectively relate the specimens in this study to taxonomic nomenclature, 11 wing characters (seven binary and four multi-state: Figure 5, Additional file 1: Table S2) were selected to differentiate the 17 subspecies in the *A. mormo* species complex recognized by [34] (Additional file 1: Table S6). Most specimens were assigned to one of *A. mormo mormo, A. mormo langei*, *A. mormo cythera*, *A. mormo tuolumnensis*, *A. mejicanus*, *A. mejicanus pueblo*, *A. virgulti*, or *A. virgulti nigrescens* (Additional file 1: Table S7). All specimens from the northern part of the range—BC, WA, OR, ID, MT, WY, SD, ND, and SK—were classified as *A. mormo mormo* on the basis of geographic origin only, due to lack of phenotypic variation and because the nominate subspecies is the only one that is considered to occur in those areas [36]. A few intermediate specimens were classified as *A.* nr. *mormo* or *A. mormo* nr. *langei*. Although *A. mormo langei* was described based on its unique phenotype [43], some individuals from populations in central California have been discovered with similar phenotypic characters, including orange scaling over the forewing discal cell spot and hindwing basal spots as well as orange scaling medially on the hindwing (characters FE1, HI2, and HG1 [Figure 5, Additional file 1: Table S2]). Specimens in this study from Mendota area (locality 15), display these characteristics and were assigned to *A. m.* nr. *langei*.

Table 2 summarizes taxonomic designations of specimens for which genetic data was obtained. Overall, there is little relationship between taxonomic designation and membership to either mtDNA clades or STRUCTURE groupings. While some subspecies display relatively narrow genetic characteristics (e.g. *A. m. langei*), most display variable signatures with both mtDNA and microsatellites. Haplotypes 0366 and h1472, which formed the sister clade to the main *A. mormo* complex lineage, assigned to *A. mejicanus pueblo*, giving 4.0% divergence between *A. mejicanus pueblo* and other *A. mejicanus* specimens. Additionally, despite their phenotypic similarity, individuals of *A. m. langei* and those assigned to *A. m.* nr. *langei* (locality 15) exhibit different mtDNA haplotypes and microsatellite groupings.

**Table 2:** **Comparison of genetic clades/clusters and taxonomic determinations: Main mitochondrial DNA clades and microsatellite clusters from STRUCTURE exhibited by specimens assigned to each taxonomic entity that was sampled from the** ***A. mormo*** **species group**

	**Mitochondrial DNA clades**										**Microsatellite clusters**					
**Name**	**OG**	**E1**	**E2**	**W1**	**W2**	**W3a**	**W3b**	**W3c**	**W3d**	**W4**	**1**	**2**	**3**	**4**	**5**	**6**
*A. mormo mormo*		X		X	X			X		X	X	X	X			
*A. mormo cythera*					X	X		X		X					X	
*A. mormo langei*				X										X		
*A. mormo tuolumensis*								X		X					X	X
*A*. nr. *mormo*						X	X	X						X	X	
*A. m.* nr. *langei*					X									X	X	
*A. virgulti virgulti*						X	X	X						X	X	X
*A. virgulti nigrescens*						X	X									
*A. mejicanus mejicanus*			X			X	X	X							X	X
*A. mejicanus pueblo*	X															

OG refers to haplotypes 0366 and h1472, which were placed as outgroups to the main eastern and western lineages.

The taxonomy and systematics of the *A. mormo* complex is unsettled (e.g. [34,39-41]), and we do not assert that these morphological designations are an answer to the delimitation of units within this taxonomic complexity. Rather, we use these designations, based on multiple interpretations and sources, to provide an objective link between our genetic data and the preexisting taxonomic nomenclature. For consistency’s sake, we follow the names used by [34], despite his warning that his arrangement of taxa within the *A. mormo* complex “should be considered tentative.” As discussed below, we hope this study can provide insight for a taxonomic revision in the future, which should include a more thorough treatment of the morphology of the group (e.g. including ventral and/or quantitative morphometric characters).

## Discussion

Our study represents the first DNA-based treatment of the *Apodemia mormo* species complex. Both maternally inherited mtDNA and biparentally inherited microsatellites show deep divergences across the Continental divide, as well as along the west coast. Within these regions, fine-scale population structure varied from being almost absent east of the Continental Divide and in the Pacific Northwest, to being highly structured in California. Mitochondrial DNA and microsatellite markers roughly agreed on population relationships, although several discontinuities were observed when comparing the two marker sets. This data provides a starting point for a reassessment of the taxonomy of the group. Accordingly, we reevaluate support for *A. m. langei* as an ESU under multiple definitions, and as a federally listed endangered species.

### Range-wide population genetic structure/phylogeography

Across its range, the *A. mormo* complex includes several distinct lineages. The most pronounced of these is the mitochondrial divergence between haplotypes 0366 and h1472, assigned to *A. mejicanus pueblo,* and the rest of the *A. mormo* complex, including other specimens assigned to *A. mejicanus*. This is the largest intraspecific divergence observed in the complex, and these populations merit further attention and focused collecting to elucidate their phylogenetic and taxonomic placement. Apart from these divergent haplotypes, both mitochondrial and microsatellite loci support deep divergences across the Continental Divide and between the Pacific Northwest and California. Although some genetic variation exists east of the Continental Divide (particularly with microsatellites), the nexus of diversity for both marker types is found in California. Here however, the concordance between mtDNA and microsatellites breaks down. Microsatellites show some populations of pure ancestry (inferred via STRUCTURE), that tend to cluster geographically, but also many admixed populations and individuals. Mitochondrial DNA also shows several “pure” clusters of geographically proximate populations, in which all individuals belong to the same clade. Some of these roughly match clusters displayed by the microsatellite dataset, although the geographic positions of the breaks between clusters overlap. California also contains many populations of mixed mitochondrial ancestry, including individuals exhibiting haplotypes from the eastern lineage (localities 37, 61, 64, and 65) and the Pacific Northwest (localities 20, 21, 23, and 25).

Populations in California exhibit higher regional population differentiation (F_ST_) than both other regions, and populations east of the Continental Divide show the lowest, despite having comparable levels of genetic diversity (observed heterozygosity) to California. Despite low vagility in these butterflies, only weak signatures of IBD are observed in the Pacific Northwest and east of the Continental Divide. Virtually no signatures of IBD were observed in California, which is unsurprising given the lack of geographic structure in microsatellite groupings. Populations in the Pacific Northwest were not only distinct, but exhibited statistically lower genetic diversity (heterozygosity) than the other regions, as well as fewer average alleles per locus. This confirms previous findings [42] but mtDNA haplotypes matching those found in the Pacific Northwest were also found in central California. Given the lower genetic diversity compared to other regions, and the presence of these mtDNA haplotypes, it is likely that the Pacific Northwest region was populated by post-glacial migrants originating in California, who experienced a population bottleneck during this process. Similar patterns have been observed in other species occupying recently glaciated areas (e.g. [44]), and this pattern is known as the leading edge hypothesis [45]. Tajima’s *D* and Fu’s *F*_*s*_ statistics potentially support recent population bottlenecks in the Pacific Northwest, but these results were not significant. East of the Continental Divide, a model of non-neutral sequence evolution was supported (i.e. population growth or purifying selection [42]), and interestingly, we also observed eastern mtDNA haplotypes in southern California, Nevada, and Mexico. The presence of these eastern haplotypes west of the Continental Divide may indicate a southwestern origin for the eastern lineage, departing from the previously hypothesized Great Plains origin presented by [42]. If the signature of non-neutral evolution of this mtDNA is due to recent population expansion in this region (which assumes neutrality for mtDNA, but see [46,47]), this would likely make this origin/divergence older than that for the Pacific Northwest. More detailed sampling between California and these two regions will help to resolve these phylogeographic hypotheses, and may reveal finer scale population structure that our sampling omits.

California’s high haplotypic and genetic diversity could be attributed to multiple phenomena. First, introgressive hybridization between neighboring populations can create patterns of admixture and facilitate movement of mtDNA haplotypes between populations/species (e.g. [48,49]), which is possible given the close relationships of members within this complex and their geographic proximity. However, under this scenario we would expect adjacent localities to share more haplotypes than distant ones (i.e. IBD), particularly in organisms that have low vagility, such as *A. mormo* [50]. Isolation by distance was virtually nonexistent and not statistically significant in California; furthermore, sites that shared identical mtDNA haplotypes were as frequently tens to hundreds of miles apart as they were adjacent. Therefore, a more likely explanation for this diversity may be retained ancestral polymorphism (i.e. incomplete lineage sorting e.g. [44,51,52]). *Apodemia mormo*’s low vagility and high habitat specificity [35,50] restricts its dispersal ability, which can lead to high levels of phylogeographic subdivision via limited gene flow [53]. This scenario, combined with high genetic diversity and differentiation observed in California, supports the hypothesis of retained ancestral polymorphism.

### Comparing genetic data and taxonomy

We found little to no concordance between our taxonomic designations and genetic placement with either mtDNA or microsatellites. The nominate subspecies of the group, *A. m. mormo*, exemplifies this point at the largest geographic scale considered in this study. From California to British Columbia and from New Mexico to Saskatchewan these butterflies can be more-or-less phenotypically identical, but exhibit deep genetic divergences in both their mitochondrial and nuclear genomes. Regionally, geographic proximity is a better predictor of genetic similarity than phenotypic appearance. However, within the three broad regions considered here, some areas exhibited stronger taxonomic/genetic/geographic correspondence than others. Northern Californian populations, for example, display higher correspondence between genetic variation and geography than in southern California (Figure 2), and even some correspondence between taxonomic assignment and mtDNA subclades (Additional file 1: Figure S1). Conversely, in southern California there is no taxonomic/genetic correspondence, and some mtDNA haplotypes (e.g. h1161, h1163, h1176) are shared between sites and species (Additional file 1: Figure S1).

It is important to reiterate that the taxonomy of the *A. mormo* complex is far from resolved, and we do not present our morphological taxonomic treatment as a resolution to this situation. Rather, this treatment aimed to provide a relatively objective comparison between our genetic data and the preexisting taxonomy. Our focus on characters associated with *A. m. langei*, and use of dorsal characters only, narrows the scope of this treatment. Given the morphological complexity within the group, we hope this genetic data can provide a novel lens with which to reevaluate the entire *A. mormo* complex, although such a reevaluation is beyond the scope of this study.

### *Apodemia mormo langei*

Due to its federally endangered status, the single population of *A. m. langei* at Antioch Dunes provides a pertinent focus for this genetic assessment of the *A. mormo* complex. Within northern California (mtDNA clade W1), individuals from Antioch Dunes form a monophyletic mtDNA subclade (Additional file 1: Figure S1), and are most closely related (<0.5% sequence divergence) to individuals from geographically proximate populations (Mt. Diablo and Vallejo, localities 10 and 12, respectively). Genetic distance estimates generated with microsatellites, on the other hand, place Antioch Dunes in closer relationship to Tumey Hills, Arroyo Bayo, and Del Puerto (localities 16, 14, and 13, respectively: Figure 4) to the south. While discordance between maternally- and biparentally-inherited markers is not uncommon [47,54], it does suggest complexity in the evolutionary history of a lineage, which can affect conservation implications. Despite their morphological similarity, *A. m. langei* is quite genetically divergent from individuals assigned to *A. m.* nr. *langei*, collected in the Mendota area (locality 15). Several individuals from this population shared some of *A. m. langei*’s distinct phenotypic characters, but exhibited divergent mtDNA (clade W2) and high levels of admixture with microsatellite loci. The habitats of these populations are not particularly similar, and the use of different *Eriogonum* hosts (Additional file 1: Table S1) makes it unlikely that convergent evolution is responsible for the phenotypic similarity.

Placed in the context of the entire species complex, *A. m. langei* is no more genetically distinctive than most populations in California, and other populations exist in this region that exhibit higher mitochondrial and nuclear divergence. Additionally, some of the morphological characteristics that earned *A. m. langei* its subspecific status are found in other populations in California. In this light, does this current data support ESU status for *A. m. langei* and, if so, under what ESU criteria? Along with many populations in northern California, the population at Antioch Dunes is reciprocally monophyletic with respect to mtDNA (Additional file 1: Figure S1), satisfying half of Moritz’s [2] criteria. This mitochondrial pattern may also support criteria such as lack of “genetic exchangeability” (no recent gene flow [6]), “long term isolation” [5], and “highly-restricted gene flow from other such lineages within the higher organization level of the species” [7], although the lack of quantitative determination of these criteria highlights their subjective nature. Microsatellite loci support *A. m. langei*’s genetic distinctiveness, but only with regional, ordination-based methods (DAPC), not with individual-based Bayesian clustering methods (STRUCTURE). Ecologically, *A. m. langei* is potentially distinctive, as its host *Eriogonum nudum* var. *psychicola* Reveal 2007 is also an endemic of the Antioch Dunes [55]. Group-wide information on *A. mormo* hosts, however, is relatively sparse (Additional file 1: Table S1), so we are reluctant to draw broad conclusions about the variability of host plants as criteria for “ecological exchangeability” [6] or general ecological distinctiveness. Below the level of an ESU, *A. m. langei*’s low vagility combined with its moderate genetic distinctiveness likely demonstrates demographic independence, a criterion of DUs [2,14]. In fact, our sole use of potentially neutral genetic loci (rather than both neutral and adaptive loci) may be more appropriate for defining DUs [15], and some argue that without both types of loci, our conclusion is inherently limited to defining a “partial ESU” rather than a “full ESU” [8]. Regardless, defining DUs quantitatively (e.g. [26]) would require increased sampling and finer-scale genetic data.

Collectively, these data provide conflicting support for *A. m. langei*’s evolutionary significance. While more data (particularly that of host plant) will provide insight into this issue, we expect that the multifarious nature of the *A. mormo* complex will undermine future determinations that *A. m. langei* is more evolutionarily divergent than other isolated populations in northern California. However, this study’s focus on evolutionary significance is not an effort to describe or compare all members of the *A. mormo* complex evolutionarily, but to address whether genetic data support *A. m. langei*’s federal listing. As we have illustrated, this question can be answered differently depending on the ESU criteria considered. While one side of this answer is that genetic data do not support *A. m. langei*’s federal listing, we believe that such an interpretation oversimplifies the role of endangered species with regard to ecosystems, particularly in the case of the Antioch Dunes. Balancing the evolutionary significance of a single species against conservation efforts for an entire ecosystem is an exceptionally difficult task, particularly when valuation of those species to the “success” of conservation is not tied to obvious economic value [56,57]. *Apodemia mormo langei* is undeniably a “flagship species” [58,59] for the Antioch Dunes ecosystem (e.g. [60,61]), but how can a value be placed on public perception or engagement? Additionally, should the momentum of several decades of conservation effort towards this ecosystem be included in a valuation? These conceptual questions are much farther-reaching than the scope of this study, however for these reasons we are not arguing for the delisting of *A. m. langei*. We hope our genetic assessment of the complex provides a foundation to reevaluate the taxonomy of the group as well as current and future conservation efforts, and we stress that these reassessments should occur in that order. Without a thorough understanding of the group’s taxonomy and systematics, conservation prioritization may not be maximally efficient. This is particularly true for invertebrates, as federally recognized units of conservation below the subspecies level are generally only applied to vertebrates (“distinct population segments” [62]).

## Conclusion

Here we used several genetic markers to assess range-wide population genetic structure of the *A. mormo* species complex. We then used this information to reassess the evolutionary significance of a federally endangered subspecies within the group. We found highly divergent lineages across the range of the *A. mormo* complex, indicating a complex evolutionary history. The nexus of genetic diversity was observed in California, where delimitation of highly structured populations agreed poorly with taxonomic designations. *Apodemia mormo langei* is no more genetically unique than various other populations of *A. mormo* in California, and even some of the morphological characteristics that earned it subspecific status are not unique. These results indicate that both the taxonomy and conservation prioritization of the *A. mormo* complex should be reassessed, both with a more fine-grained genetic survey and a greater focus on locally adapted phenotypic traits.

## Methods

### Sampling

A total of 548 specimens of *Apodemia* and outgroups were obtained from six principal sources (Additional file 1: Table S8). Specimens of *A. mormo langei* from Antioch Dunes NWR were collected in 1997 under US Fish & Wildlife Service collection permit PRT-832200. Collection, vouchering, and preservation of specimens differed among sources (Additional file 1: Table S1). We sequenced the full mitochondrial *cytochrome oxidase subunit I* (COI) gene (1498 base pairs) and up to five microsatellite loci were scored from as many specimens as possible. The principal exceptions were 82 specimens from Opler, Davenport *et al.* (Source #3, Additional file 1: Table S8). The only genetic data available to us from those samples were the 648 base pairs of the “barcode region” of the COI gene, which were obtained by PO from the Barcode of Life Database (BOLD). In total, sampling of the *A. mormo* species complex spanned 69 geographic locations in 12 states and two provinces in three countries (Additional file 1: Table S1), comprising most of the known range.

All vouchered specimens were photographed, with the exception of 38 specimens from Grasslands National Park, SK, which were preserved in ethanol. Dorsal-view photographs of most specimens were taken by BP with an 8.0 megapixel Nikon Coolpix 8400 mounted on an Olympus SZX16 dissecting microscope illuminated with a fiber-optic light source. Images are available at the DNA voucher site of the Strickland Museum of Entomology (http://www.biology.ualberta.ca/uasm/Vouchers/index.html). Dorsal images of the specimens from Opler, Davenport *et al.* (Source #3, Additional file 1: Table S8) were obtained by PO from BOLD (www.boldsystems.org).

### DNA extraction

Several methods of DNA extraction were used. DNA was extracted from the samples collected by Proshek *et al*., Powell, and Davenport (sources #1, 4, 5, Additional file 1: Table S8) from two legs (or leg fragments and antennae, if the specimen was in poor condition) using the DNeasy Tissue Extraction Kit (Qiagen, Valencia, CA). DNA from the specimens collected by Sperling, Powell *et al*. (Additional file 1: Table S8) was extracted from the thorax using a phenol-chloroform method as outlined in [63]. Sequences for the samples collected by Opler, Davenport et al. (Additional file 1: Table S8) were obtained from the Canadian Centre for DNA Barcoding (Guelph, ON) (www.dnabarcoding.ca) [64], but we did not have access to these DNA extractions. DNA was extracted from the samples collected by Crawford and Desjardins (Additional file 1: Table S8) from wing clips as in [65].

### mtDNA sequencing

The mitochondrial gene COI was sequenced in its entirety for as many specimens as possible. In total, 469 sequences of 1498 base pairs in length were obtained. For the samples collected by Sperling, Powell *et al*. (Additional file 1: Table S8), 398 base pairs of the gene were initially sequenced using the primers Jerry (C1-J-2183) [66] and K741 (C1-N-2578a) [67], following [67]. The rest of the gene was later amplified in two fragments using the primer pairs LCO1490 to HCO2198 [68] and BrianXXVII to Pat [66]. For all other samples, the COI gene was sequenced in two fragments: LCO to HCO and Jerry to Pat, unless chromatogram signal was poor, in which case the internal primers Jerry and Mila (MilaX, GATAGTCCTGTAAATAATGG, for samples from west of the Rocky Mountains and MilaXI, GATAATCCTGTAAATAATGG, for samples from east of the Rocky Mountains) and BrianXXVII and Pat were used. The polymerase chain reaction and cycle sequencing protocols are given in detail in [42]. Chromatograms were checked for signal quality in Lasergene (DNASTAR, Madison, WI). Priming sites were manually removed and sequences were manually aligned in Mesquite 2.72 [69].

### Microsatellite development, amplification and genotyping

We isolated and characterized six novel microsatellite loci from two libraries. Details of library development and locus amplification are given in [42]. Genotyping was carried out in GeneMapper (Applied Biosystems, Foster City, CA). We obtained genotype scores for a total of 447 samples from all sampling sources except Source #3 (Additional file 1: Table S8). Amplification success was not consistent across sampling areas. Locus E7 did not amplify in individuals west of the Rockies, and locus M2 did not amplify for individuals east of the Rockies (one exception: the two samples from Sonora, Mexico did not amplify at E7 but one did amplify at M2). All samples, therefore, were genotyped at a maximum of five loci. Sixty samples were genotyped at four loci and 52 at three loci. Samples that amplified less than three loci were not used.

### Sequence analysis

There was a total of 205 unique COI haplotypes: 157 haplotypes were 1498 base pairs in length, and 48 “barcode” haplotypes 648 base pairs in length. Haplotypes were only considered unique if there was at least one base substitution relative to all other haplotypes. Missing base pairs were scored as “N” (missing). All 1498-base pair COI haplotypes from the specimens from Sperling, Powell *et al*. (Additional file 1: Table S8) had 18 missing base pairs from the middle of the haplotypes where the internal primers overlapped; all other 1498-base pair COI haplotypes had 11 missing base pairs at the same location.

A maximum-likelihood phylogenetic analysis of the COI sequences was performed in Garli 1.0 [70] under the TPM2uf + I + G model, which was selected by jModelTest 0.1.1 [71,72] as the most likely model for our data under the AIC, AICc, and BIC model selection criteria. For the best-tree analysis, rates were constrained so that r[AC] = r[AT], r[AG] = r[CT], and r[CG] = r[GT]. The rate parameters, base frequencies, proportion of invariable sites, and gamma shape parameter were estimated during analysis. Twenty-five search replicates were performed to find the best tree. Two hundred fifty bootstrap replicates were also performed under the same model, except with parameters fixed at the following values: r[AC] = r[AT] = 4.6640; r[AG] = r [CT] = 36.8988; r[CG] = r [GT] = 1.000; eqA = 0.3248, eqC = 0.1293, eqG = 0.1129, eqT = 0.4330; proportion invariable sites = 0.4780; and gamma shape parameter = 0.3120. *Calephelis wrighti* and *Emesis emesia* were selected as outgroups, both of which are members of the subfamily Riodinidae; the former in the Riodinini and the latter, like *Apodemia*, is *incertae sedis* [73]. We tested for neutral sequence evolution by calculating Tajima’s *D* [74] and Fu’s *F*_*s*_ statistics [75] in Arlequin v3.5 [76]. Positive values for these statistics indicate potential population bottlenecks or balancing/overdominant selection, while negative values indicate potential population size increase or purifying selection [77]. These statistics were calculated globally, regionally (Pacific Northwest, east of the divide, and California), and for each population in California (Tajima’s *D* and Fu’s *F*_*s*_ were calculated for the other two regions in [42]).

### Microsatellite analysis

The program STRUCTURE [78] was used to determine the smallest number of genetic clusters that maximized Hardy-Weinberg equilibrium. We tested K values (number of genetic clusters) between 2 and 20 with seven replications each, using the admixture model and correlated allele frequencies. 40,000 burn-in generations and 240,000 post burn-in generations were run. ∆K was calculated following the Evanno *et al.* [79] method using STRUCTURE HARVESTER [80], and CLUMPP was used to average multiple runs of each K value [81]. STRUCTURE was run with and without population information as a prior, but results did not differ significantly with the addition of population information. To assess microsatellite clustering without assuming Hardy-Weinberg and gametic equilibrium, we conducted discriminant analysis of principal components (DAPC [82]). This method maximizes between- and minimizes within-group variability by conducting a principal components analysis on genetic data, before submitting those principal components to a discriminant analysis. DAPC was implemented in R v3.0.1 [83] using *adegenet* v1.3.1 [84]. *adegenet*’s *find.clusters* function was used to estimate the ideal value of K (default parameters, retaining all principal components), and *optim.a.score* was used to estimate the ideal number of principal components to retain in the final discriminant analysis (using default settings and 25 full simulations).

Pair-wise population differentiation (F_ST_) and summary statistics (heterozygosity, number of alleles per locus) were calculated using GENEPOP v4.2 [85,86] and the Excel Microsatellite Toolkit v3.1.1 [87], respectively. To test for signatures of isolation by distance (IBD [88]), matrices of population-level, standardized genetic differentiation [F_ST_/(1-F_ST_)] [89] and geographic distance between localities were constructed using GENEPOP v4.2 and the Geographic Distance Matrix Generator v1.2.3 [90], respectively. The degree of correlation between these matrices was evaluated with a Mantel test [91] implemented in GENEPOP v4.2 using 9,999 randomizations. All measures of population differentiation, descriptive statistics, and tests for IBD were evaluated for the entire microsatellite dataset, as well as for each main geographic region (Pacific Northwest, east of the divide, and California) separately. To compare regional values of heterozygosity, non-parametric Wilcox-rank sum tests were conducted in R [83]. A Bonferroni correction was used for all multiple pair-wise tests.

### DNA sequence & microsatellite comparison

In order to provide a more direct comparison between microsatellites and mtDNA, a phylogenetic tree was generated using 154 unique mtDNA haplotypes from samples for which we also had microsatellite genotypes. We generated a maximum-likelihood tree in Garli 1.0 [70] under the GTR + I + G model selected by jModelTest 0.1.1 [71,72] as the most likely for our data under both the AIC and hLRT criteria. All parameters were estimated during analysis. Twenty-five search replicates were performed to find the best tree. Two hundred fifty bootstrap replicates were also performed under the same conditions. All trees were unrooted. The terminal tips were manually condensed into simplified groupings that approximated the populations from which the haplotypes were sampled, in order to compare the topology of the tree to a tree generated by analysis of microsatellite genetic distances in *a priori* populations. The program Poptree2 [92] was used to generate a neighbor-joining tree based on the Da genetic distances [93] of population microsatellite allele frequencies within *a priori* populations. 1000 bootstrap replicates were performed.

### Wing characters

Eleven wing characters were selected to objectively relate the genetic data produced in this study to the existing taxonomic nomenclature of the 17 subspecies in the *A. mormo* species complex recognized by [34] (Additional file 1: Table S2). All subspecies in the *A. mormo* complex (as documented by [34]) were included in this morphological treatment, despite our genetic focus on only part of the complex. Characters were selected based on the original taxonomic descriptions (Additional file 1: Table S3), further descriptions of character variation in [35], examination of photographs in the Butterflies of America website [94], and geographic considerations. Images of type specimens were examined when available; otherwise images of several representative specimens were used. Characters were limited to the dorsal side only and chosen to be independent of specimen size or interpretation of shades of color. This allowed specimens to be scored with these characters based only on a dorsal-view photograph, irrespective of light source or camera settings, and without need of a scale bar.

### Availability of supporting data

The sequence dataset supporting the results of this article are available in the GenBank repository (Additional file 1: Table S9), and other data files are available upon request.

## Additional files

Additional file 1: Figure S1. Maximum-likelihood trees of all unique COI haplotypes. **Table S1.** Collection locality data. **Table S2.** Wing characters and descriptions. Table S3. Species descriptions. **Table S4.** Tajima’s D and Fu’s F statistics for Californian populations. **Table S5.** Pair-wise F_ST_ and heterozygosity values. **Table S6.** Diagnostic wing characters. **Table S7.** Morphological dataset. **Table S8.** Summary of the six major sources of specimens. **Table S9.** GenBank accession numbers.
