# Supplementary material for: Genetic evaluation of the evolutionary distinctness of a federally endangered butterfly, Lange’s Metalmark
Source: BMC Evol Biol. 2015 Apr 25;15:73. doi: 10.1186/s12862-015-0354-9 (PMC4427997; doi:10.1186/s12862-015-0354-9)
Supplement: Additional file 1: Figure S1. — Maximum-likelihood trees of all unique COI haplotypes. Table S1. Collection locality data. Table S2. Wing characters and descriptions. Table S3. Species descriptions. Table S4. Tajima’s D and Fu’s F statistics for Californian populations. Table S5. Pair-wise FST and heterozygosity values. Table S6. Diagnostic wing characters. Table S7. Morphological dataset. Table S8. Summary of the six major sources of specimens. Table S9. GenBank accession numbers. [file 12862_2015_354_MOESM1_ESM.doc]

**Additional file 1**

Additional file 1: Figure S1 (previous page). Maximum-likelihood trees of all unique COI haplotypes: Tree generated in GARLI 1.0 (Zwickl 2006). (A) Lineage of haplotypes from east of the Rockies; (B) Lineage of haplotypes from west of the Rockies (Additional file 1: Table S1). Haplotype names of four digits indicate 648 base pairs haplotypes; haplotype names of three digits indicate haplotypes of 1498 base pairs. Numbers above branches or pointing to nodes indicate bootstrap support based on 250 repetitions. Scale bars proportional to changes per site. “Taxa” column indicates the name assigned to the specimens represented by each haplotype: mor = *mormo*, mej = *mejicanus*, vir = *virgulti*, nig = *virgulti nigrescens*, cyt = *mormo cythera*, tuo = *mormo tuolumnensis*, lan = *mormo langei*, nr. mor = nr. *mormo*, nr. lan = *mormo* nr. *langei*. N indicates the number of samples represented by each haplotype; a number in parentheses indicates the number of samples in that haplotype represented in the Structure groupings, if different. Structure groupings indicate the number of samples represented by each haplotype that are found in each the six Q groupings found by analysis of microsatellite allele frequencies in Figure 2. Location refers to state/province and locality number(s) for each haplotype.

Additional file 1: Table S1. Collection locality data: region, location, locality number (#, see Figure 2 or S1), taxonomic designation^a^, host plant present, collection date, collector(s), latitude, longitude, sample size for microsatellites (SSR) and mitochondrial DNA (mtDNA), and mtDNA haplotypes found at each locality.

| Region | Location | # | Taxonomy^a^ | *Eriogonum* present | Coll. Date | Collector | Lat. (deg) | Long. (deg) | SSR (n) | mtDNA (n) | mtDNA haplotypes (n) |
| --- | --- | --- | --- | --- | --- | --- | --- | --- | --- | --- | --- |
| CAN: BC | near Keremeos: site N1 | 01 | A. mor. | *E. niveum* | 23-Aug-2008 | L. Crawford, S. Desjardins | 49.26469 | -119.82383 | 5 | 10 | h356 |
| CAN: BC | near Keremeos: site C1 | 01 | A. mor. | *E. niveum* | 12-Aug-2008 | L. Crawford, S. Desjardins | 49.20787 | -119.8246 | 5 | 10 | h356 |
| CAN: BC | near Keremeos: site W8 | 01 | A. mor. | *E. niveum* | 17-Aug-2008 | L. Crawford, S. Desjardins | 49.20681 | -119.85524 | 3 | 5 | h356 |
| CAN: BC | near Keremeos: site W6 | 01 | A. mor. | *E. niveum* | 5-Sep-2008 | B. Proshek, S. Desjardins | 49.20430 | -119.86720 | 10 | 10 | h350, h356 |
| CAN: BC | near Keremeos: site E2 | 01 | A. mor. | *E. niveum* | 18-Aug-2008 | L. Crawford, S. Desjardins | 49.17759 | -119.7803 | 8 | 9 | h350, h356 |
| USA: WA | Shanker's Bend, Similkameen River Cyn., W of Oroville | 02 | A. mor. | *E. niveum* | 22-Aug-2008 | L. Crawford, S. Desjardins | 48.97314 | -119.508208 | 5 | 8 | SHK02, 350 |
| USA: WA | Toats Coulee Ck., W of Sinlahekin Ck., S of Palmer Lake | 03 | A. mor. | *E. niveum* | 6-Sep-2008 | B. Proshek | 48.83255 | -119.67781 | 10 | 10 | h350, h371 |
| USA: WA | Bluffs E of the Okanogan River at Riverside | 04 | A. mor. | *E. niveum* | 4-Sep-2008 | B. Proshek | 48.50761 | -120.46909 | 10 | 11 | h350, h352, h356, h358 |
| USA: WA | Umtanum Ck off Hwy 281, S of Ellensburg | 05 | A. mor. | *E. niveum* | 3-Sep-2008 | B. Proshek | 46.85023 | -120.48841 | 8 | 9 | h341, h342, h343, h344, h345, h346 |
| USA: ID | Bluffs E of Graves Creek Rd., 8 km S of Cottonwood | 06 | A. mor. | *E. niveum* | 30-Aug-2008 | B. Proshek | 45.97489 | -116.36036 | 11 | 11 | h318, 321, h323, 326 |
| USA: OR | Just N of junction US 395 & OR 74 | 07 | A. mor. | *E. niveum* | 1-Sep-2008 | B. Proshek | 45.46236 | -118.98676 | 9 | 12 | h329, h330, h332,h 335 |
| USA: CA | Hull Mountain, Lake/Mendocino Co. line | 08 | A. mor. | *E. umbellatum* | 19-Sep-1995 | J. Powell | 39.52 | -122.94 | 10 | 10 | h046, 053, 054, 055 |
| USA: CA | 2 mi. W of Ladoga, Colusa Co. | 09 | A. mor. | *E. umbellatum* var. *bahiiforme* | 17-Sep-1997 | J. Powell, F. Sperling | 39.09 | -122.24 | 10 | 10 | h081, h082, h083, 085 |
| USA: CA | NE Vallejo, St. John's Mine, Solano Co. | 10 | A. mor. | possibly *E. nudum* | 21-Aug-1997 | J. Powell, F. Sperling | 38.14 | -122.20 | 10 | 10 | h066 |
| USA: CA | Antioch Dunes, Sardis Section, Contra Costa Co. | 11 | A. mor. lan. | *E. nudum* var. *psychicola* | 18-Sep-1997 | F. Sperling, R. Reed, A. Cognato | 38.02 | -121.80 | 9 | 10 | 091, h092, 093, h094, h096 |
| USA: CA | Below Mount Diablo's main summit, Contra Costa Co. | 12 | A. mor. | *E. umbellatum* var. *bahiiforme* | 21-Aug-1997 | J. Powell, F. Sperling | 37.88 | -121.91 | 10 | 10 | h056, h058, h059, 060, h061 |
| USA: CA | Del Peurto Canyon, Stanislaus Co. | 13 | A. mor. | *E. nudum* var. *bahiiforme* | 14-Sep-1995 | J. Powell | 37.42 | -121.35 | 10 | 10 | h036, h038, 039, h040 |
| USA: CA | Arroyo Bayo, Santa Clara Co. | 14 | A. mor. | *E. umbellatum* var. *bahiiforme* | 14-Sep-1995 | J. Powell | 37.38 | -121.57 | 10 | 10 | h026, 027, 028, 029, h031, 032, 033, 035 |
| USA: CA | Mendota area, Monocline Ridge, Fresno Co. | 15 | A. mor. nr. lan. | *E. latifolium* var. *indictum* | 11-Sep-1995 | J. Powell, F. Sperling | 36.75 | -120.38 | 10 | 10 | 001, h002, h003, 004, 006, 007 |
| USA: CA | Tumey Hills, Fresno Co. | 16 | A. mor. | *E. latifolium* var. *indictum* | 11-Sep-1995 | J. Powell, F. Sperling | 36.61 | -120.67 | 10 | 10 | h013, h017, 018, 019, 020, 022 |
| USA: CA | Limestone Camp, Kern Cyn, mtn. 99, Tulare Co | 17 | A. cyt. tuo. | *E. fasciculatum* | 25-Aug-2002 | K. Davenport | 36.09 | -118.45 | 1 | 1 | 425 |
| USA: CA | Monterey Co. Parkfield Grade | 18 | A. mej. | no data | 30-Aug-2000 | K. Davenport | 35.985 | -120.474 | 0 | 1 | h1471 |
| USA: CA | Goldledge Camp, Kern Cyn, Tulare Co. | 19 | A. cyt. tuo. | *E. fasciculatum* | 25-Aug-1989 | K. Davenport | 35.95 | -118.46 | 0 | 0 | - |
| USA: CA | Nine Mile Cyn., Inyo Co. | 20 | A. mej. | *E. inflatum* | 22-Jul-2008 | K. Davenport | 35.837 | -117.906 | 0 | 1 | h1142 |
| USA: CA | Nine Mile Cyn., Inyo Co. | 20 | A. mej. | *E. inflatum* | 05-Aug-2008 | K. Davenport | 35.837 | -117.906 | 0 | 1 | h1142 |
| USA: CA | Nine Mile Cyn., Inyo Co. | 20 | A. cyt. tuo. | *E. umbellatum* | 23-Aug-2008 | K. Davenport | 35.837 | -117.906 | 0 | 1 | 1160 |
| USA: CA | N of Hospital Flat, Kern Rd. Cyn., Tulare Co. | 21 | A. cyt. | *E. fasciculatum* | 15-Sep-2002 | K. Davenport | 35.79 | -118.44 | 1 | 1 | h412 |
| USA: CA | S. Corral Ck, Tulare Co. | 21 | A. cyt., A. cyt. tuo. | *E. fasciculatum* | 30-Oct-2002 | K. Davenport | 35.79 | -118.44 | 4 | 4 | h412, 414 |
| USA: CA | Kern Cyn., Corral Ck., Tulare Co. | 21 | A. cyt., A. cyt. tuo. | *E. fasciculatum* | 5-Oct-2002 | K. Davenport | 35.79 | -118.44 | 3 | 3 | h412, 415, 419 |
| USA: CA | Kern Cyn, S of Corral Ck., Tulare Co. | 21 | A. cyt., cyt. tuo. | *E. fasciculatum* | 6-Nov-2002 | K. Davenport | 35.79 | -118.44 | 3 | 3 | h412, 417 |
| USA: CA | Corral Ck., 1 mi. S of Kern Cyn., Tulare Co. | 21 | A. cyt. tuo. | *E. fasciculatum* | 14-Nov-2001 | K. Davenport | 35.79 | -118.44 | 1 | 1 | 421 |
| USA: CA | S of Corral Ck., Kern Cyn., Tulare Co. | 21 | A. cyt., A. cyt. tuo. | *E. fasciculatum* | 23-Oct-2002 | K. Davenport | 35.79 | -118.44 | 3 | 3 | h412, 429 |
| USA: CA | 1.5 mi. S. of Kernville, Kern Co. | 22 | ***** | *E. wrightii* possibly *E. fasciculatum* | 09-Oct-2004 | K. Davenport | 35.734 | -118.456 | 0 | 5 | 1174, 1175, h1176, h1471, h1163 |
| USA: CA | S. of Kernville E. side - Greenhorn Mountains | 22 | A. nr. mor. | *E. wrightii* | 22-Oct-2005 | K. Davenport | 35.734 | -118.456 | 0 | 4 | h1163 |
| USA: CA | 2 mi S. of Kernville | 22 | A. vir. | *E. wrightii* | 12-Nov-2007 | K. Davenport | 35.734 | -118.456 | 0 | 6 | h1176 |
| USA: CA | Greenhorn Mountains, Old State Rd., Kern Co. | 23 | A. cyt. | *E. umbellatum* | 21-Aug-2006 | K. Davenport | 35.714 | -118.517 | 0 | 2 | h1186, 1187 |
| USA: CA | Greenhorn Mountains, Old State Rd., Kern Co. | 23 | A. cyt. | *E. umbellatum* | 21-Aug-2004 | K. Davenport | 35.714 | -118.517 | 0 | 2 | h1186 |
| USA: CA | Lake Isabella Sierra Highway, Stine Cove, Kern Co. | 24 | A. mej., A. nr. mor. | *E. wrightii* | 20-Sep-2008 | K. Davenport | 35.680 | -118.410 | 0 | 3 | h1176, 1159, 1471 |
| USA: CA | Walker Pass, Kern Co. | 25 | A. cyt. tuo. | *E. fasciculatum* | 31-Aug-2002 | K. Davenport | 35.66 | -118.03 | 1 | 1 | 422 |
| USA: CA | Jawbone Cyn, Kern Co. | 26 | A. mej. | *E. inflatum* | 13-Apr-2000 | J. Powell | 35.41 | -118.10 | 2 | 2 | h431A, h431B |
| USA: CA | Sand Cyn., Tehachapi Mountains, Kern Co. | 27 | A. vir. | *E. fasciculatum* | 13-May-2001 | K. Davenport | 35.309 | -118.429 | 0 | 2 | 1154, 1167 |
| USA: CA | Last Chance Cyn, Kern Co. | 28 | A. mej. | *E. inflatum* | 10-Apr-2000 | J. Powell | 35.29 | -118.46 | 4 | 5 | h431A, h431B |
| USA: CA | Tehachapi Mountains, Willow Springs Rd., Kern Co. | 29 | A. mej., A. nr. mor. | *E. inflatum* | 03-May-2008 | K. Davenport | 35.073 | -118.398 | 0 | 2 | h1155 |
| USA: CA | Bates Canyon, Santa Barbara Co. | 30 | A. cyt. | *E. fasciculatum* | 08-Sep-2002 | K. Davenport | 34.953 | -119.907 | 0 | 2 | h1165 |
| USA: CA | Dry & Santa Barbara Canyons, Santa Barbara Co. | 31 | A. cyt. | *E. fasciculatum* | 21-Aug-2004 | K. Davenport | 34.947 | -119.691 | 0 | 1 | 1189 |
| USA: CA | Valle Vista Camp, Kern Co. | 32 | A. cyt. | *E. wrightii* | 05-Sep-2006 | K. Davenport | 34.878 | -119.340 | 0 | 1 | h1163 |
| USA: CA | McGill Camp Mount Pinos, Kern Co. | 33 | A. cyt. | *E. umbellatum* | 05-Sep-2006 | K. Davenport | 34.819 | -119.095 | 0 | 2 | h1163 |
| USA: CA | Old Ridge Route, L.A. Co. | 34 | A. cyt., A. cyt. tuo. | *E. fasciculatum* | 02-Sep-2007 | K. Davenport | 34.605 | -118.692 | 0 | 2 | h1161 |
| USA: CA | Lake Hughes Rd. E - Lake Castaic, L.A. Co. | 35 | A. vir. | *E. fasciculatum* | 07-Oct-2007 | K. Davenport | 34.596 | -118.563 | 0 | 1 | 1149 |
| USA: CA | Lake Hughes Road Warm Springs, L.A. Co. | 35 | A. vir., A. cyt. | *E. fasciculatum* | 14-Oct-2007 | K. Davenport | 34.596 | -118.563 | 0 | 4 | 1148, 1152, h1161 |
| USA: CA | Lake Hughes Road Warm Springs, L.A. Co. | 35 | A. vir. | *E. fasciculatum* | 30-Sep-2007 | K. Davenport | 34.596 | -118.563 | 0 | 1 | 1150 |
| USA: CA | Lake Hughes Road Warm Springs, L.A. Co. | 35 | A. vir. | *E. fasciculatum* | 04-Oct-2007 | K. Davenport | 34.596 | -118.563 | 0 | 1 | 1151 |
| USA: CA | Three Points, Los Angeles Co. | 36 | A. vir. | No data | 28-Aug-1999 | K. Davenport | 34.343 | -117.982 | 0 | 3 | h1161 |
| USA: CA | Rock Corral, San Bernardino Co. | 37 | A. mej. | *E. inflatum* | 11-Apr-2000 | J. Powell | 34.27 | -116.47 | 2 | 2 | 435B, 435C |
| USA: CA | near Onyx Summit, San Bernardino National Forest | 38 | A. nr. mor. | No data | 26-Jun-1998 | S. Cho, J. Powell, F. Sperling | 34.20 | -116.81 | 10 | 10 | 125, 126, 127, h128, 130 |
| USA: CA | Mil Potrero Rd., Ventura Co. | 39 | A. cyt. | No data | 05-Sep-2006 | K. Davenport | 34.140 | -118.881 | 0 | 1 | 1476 |
| USA: CA | Colton - Pepper St. & Slover Ave., San Bernardino Co. | 40 | A. vir. nig. | *E. fasciculatum* | 12-Apr-2006 | K. Davenport | 34.074 | -117.314 | 0 | 5 | 1180, 1181, 1182, 1183, 1184 |
| USA: CA | Camp Pendleton, San Diego Co. | 41 | A. vir. | no data | 14-Oct-1997 | D. Rubinoff | 33.32 | -117.43 | 5 | 5 | 111, 112, 113, h114, 115 |
| USA: CA | Camp Pendleton, San Diego Co. | 41 | A. vir. | no data | 15-Oct-1997 | D. Rubinoff | 33.32 | -117.43 | 1 | 1 | 121 |
| USA: CA | Camp Pendleton, San Diego Co. | 41 | A. vir. | no data | 27-Oct-1997 | D. Rubinoff | 33.32 | -117.43 | 4 | 4 | h114, 116 |
| USA: CA | Camp Pendleton, San Diego Co. | 41 | A. vir. | no data | 27-Oct-1998 | D. Rubinoff | 33.32 | -117.43 | 1 | 1 | 120 |
| USA: CA | Point Loma, Cabrillo National Monument, San Diego | 42 | A. vir. | no data | ?-May-1997 | D. Rubinoff | 32.67 | -117.24 | 10 | 10 | h071, 072, h073, 079 |
| CAN: SK | West Block, Grasslands National Park: Laounenan | 43 | A. mor. mor. | *E. pauciflorum* | 15-Aug-2008 | B. Proshek, M. Fairbairn | 49.20603 | -107.56911 | 6 | 5 | h156, h157 |
| CAN: SK | West Block, Grasslands National Park: Timbergulch | 43 | A. mor. mor. | *E. pauciflorum* | 15-Aug-2008 | B. Proshek, M. Fairbairn | 49.19856 | -107.50081 | 4 | 4 | h139, h156 |
| CAN: SK | West Block, Grasslands National Park: Police Coulee | 43 | A. mor. mor. | *E. pauciflorum* | 15-Aug-2008 | B. Proshek, M. Fairbairn | 49.17960 | -107.52509 | 4 | 4 | h156, h166 |
| CAN: SK | West Block, Grasslands National Park: Police Coulee | 43 | A. mor. mor. | *E. pauciflorum* | 17-Aug-2007 | A. Henderson | 49.17960 | -107.52509 | 3 | 3 | h139, h156 |
| CAN: SK | West Block, Grasslands National Park: Timmons Coulee | 43 | A. mor. mor. | *E. pauciflorum* | 15-Aug-2008 | B. Proshek, M. Fairbairn | 49.18259 | -107.54510 | 4 | 4 | h135, h156, 171 |
| CAN: SK | West Block, Grasslands National Park: Timmons Coulee | 43 | A. mor. mor. | *E. pauciflorum* | 16-Aug-2007 | (unknown) | 49.18259 | -107.54510 | 2 | 2 | h156 |
| CAN: SK | West Block, Grasslands National Park: Mid 70 Mile | 43 | A. mor. mor. | *E. pauciflorum* | 15-Aug-2008 | B. Proshek, M. Fairbairn | 49.18724 | -107.66578 | 4 | 4 | h137, h156, h157 |
| CAN: SK | West Block, Grasslands National Park: Broken Hills | 43 | A. mor. mor. | *E. pauciflorum* | 16-Aug-2008 | B. Proshek | 49.15049 | -107.56326 | 8 | 8 | h137, h139, h178, h180 |
| CAN: SK | West Block, Grasslands National Park: Broken Hills | 43 | A. mor. mor. | *E. pauciflorum* | 20-Aug-2007 | A. Henderson | 49.15049 | -107.56326 | 4 | 4 | h137, h180 |
| CAN: SK | West Block, Grasslands National Park: 70 Mile | 43 | A. mor. mor. | *E. pauciflorum* | 19-Aug-2008 | K. Fink, C. Dutchak | 49.20295 | -107.65740 | 7 | 7 | h135, h137, h139, h156, h180 |
| CAN: SK | West Block, Grasslands National Park: Broken Hills | 43 | A. mor. mor. | *E. pauciflorum* | 21-Aug-2007 | A. Henderson | 49.20295 | -107.65740 | 3 | 3 | h137, h156, 400 |
| CAN: SK | West Block, Grasslands National Park: S 70 Mile | 43 | A. mor. mor. | *E. pauciflorum* | 29-Aug-2008 | A. Henderson | 49.15450 | -107.68015 | 3 | 3 | h139, h156 |
| CAN: SK | West Block, Grasslands National Park: S Gillespie | 43 | A. mor. mor. | *E. pauciflorum* | 29-Aug-2008 | A. Henderson | 49.01783 | -107.27961 | 1 | 1 | h137 |
| CAN: SK | West Block, Grasslands National Park: S Gillespie | 43 | A. mor. mor. | *E. pauciflorum* | 12-Aug-2008 | A. Henderson | 49.01783 | -107.27961 | 4 | 4 | h137, h166 |
| CAN: SK | West Block, Grasslands National Park: S Gillespie | 43 | A. mor. mor. | *E. pauciflorum* | 16-Aug-2007 | A. Henderson | 49.01783 | -107.27961 | 1 | 1 | 405 |
| CAN: SK | West Block, Grasslands National Park: N Gillespie | 43 | A. mor. mor. | *E. pauciflorum* | 15-Aug-2007 | A. Henderson | 49.12839 | -107.25547 | 1 | 1 | h137 |
| CAN: SK | East Block, Grasslands National Park: 1 | 44 | A. mor. mor. | *E. pauciflorum* | 11-Aug-2008 | A. Henderson, C. Dutchak, B. Proshek | 49.04011 | -106.57832 | 6 | 6 | h135, 136, h137, h139, 140 |
| CAN: SK | East Block, Grasslands National Park: 1 | 44 | A. mor. mor. | *E. pauciflorum* | 12-Aug-2008 | A. Henderson, M Fairbairn | 49.04011 | -106.57832 | 1 | 1 | h137 |
| CAN: SK | East Block, Grasslands National Park: 1 | 44 | A. mor. mor. | *E. pauciflorum* | 13-Aug-2008 | C. Dutchak, K. Fink | 49.04011 | -106.57832 | 2 | 2 | h135, h137 |
| CAN: SK | East Block, Grasslands National Park: 2 | 44 | A. mor. mor. | *E. pauciflorum* | 12-Aug-2008 | A. Henderson, M Fairbairn | 49.05735 | -106.57436 | 4 | 4 | h135, h137 |
| CAN: SK | East Block, Grasslands National Park: 3 | 44 | A. mor. mor. | *E. pauciflorum* | 12-Aug-2008 | B. Proshek | 49.01677 | -106.54233 | 6 | 6 | h137, h139 |
| CAN: SK | East Block, Grasslands National Park: 4 | 44 | A. mor. mor. | *E. pauciflorum* | 12-Aug-2008 | B. Proshek | 49.02457 | -106.54509 | 2 | 2 | h137 |
| USA: MT | Dry bluffs just S of Hinsdale | 45 | A. mor. mor. | *E. pauciflorum* | 17-Aug-2008 | B. Proshek | 48.37247 | -107.09170 | 10 | 10 | h139, h156, h180 |
| USA: MT | Missouri River bluffs E of Hwy 16, S of Culbertson | 46 | A. mor. mor. | *E. pauciflorum* | 18-Aug-2008 | B. Proshek | 48.12879 | -104.47260 | 16 | 16 | h196, h197, 198, 199, h202, 206, h207, 208 |
| USA: MT | E of Sidney, near junction of SR 23 and Hwy 261 | 47 | A. mor. mor. | *E. pauciflorum* | 19-Aug-2008 | B. Proshek | 47.66215 | -104.13214 | 7 | 7 | 212, 213, h214, 216, 217, 218 |
| USA: MT | Co. Rd 467, S of Circle | 48 | A. mor. mor. | *E. pauciflorum* | 20-Aug-2008 | B. Proshek | 47.31305 | -105.59655 | 5 | 5 | h219, 220, h223 |
| USA: MT | Badlands just E of Makoshika SP, near Glendive | 49 | A. mor. mor. | *E. pauciflorum* | 21-Aug-2008 | B. Proshek | 47.04881 | -104.66299 | 30 | 29 | h197, h202, h225, h226, 228, 232, h237, 240 |
| USA: ND | Burning Coal Vein Campground, NW of Amidon | 50 | A. mor. mor. | *E. pauciflorum* | 23-Aug-2008 | B. Proshek | 46.59727 | -103.44460 | 10 | 10 | h225, h254, 255, 256, 258, h259, 260, 261 |
| USA: MT | Dry bluffs 7 mi N of Laurel on Hwy 532 | 51 | A. mor. mor. | *E. pauciflorum* | 28-Aug-2008 | B. Proshek | 45.80759 | -108.83447 | 11 | 11 | h287, h307, 309, h311 |
| USA: MT | Hollenbeck Draw, 5 mi S of Belfrey | 52 | A. mor. mor. | *E. pauciflorum* | 27-Aug-2008 | B. Proshek | 45.07076 | -109.03241 | 21 | 21 | 286, h287, 295, h297, 298, 300 |
| USA: SD | McNenny Fish Hatchery, near Spearfish | 53 | A. mor. mor. | *E. pauciflorum* | 25-Aug-2008 | B. Proshek | 44.56734 | -104.01652 | 10 | 10 | h207, h259, h265 |
| USA: WY | Upper Powder River Rd., exit 88 off US 90W | 54 | A. mor. mor. | no data | 26-Aug-2008 | B. Proshek | 44.22189 | -106.15839 | 12 | 12 | h223, h226, 274, h275, 278, h281, 283, 285 |
| USA: CO | 1 mile north-northeast of Virginia Dale, Larimer Co. | 55 | A. mej. pue. | no data | 09-Aug-2007 | P. Opler | 40.955 | -105.334 | 0 | 1 | 0366 |
| USA: CO | Golden Gate S.P. Knott Cr., Jefferson Co. | 56 | A. nais | no data | 04-Jul-2004 | P. Opler | 39.819 | -105.378 | 0 | 4 | h1293, 1295 |
| USA: CO | Mt Zion, Jefferson Co. | 57 | A. nais | no data | 05-Jul-1992 | R. E. Stanford | 39.744 | -105.242 | 0 | 1 | h1293 |
| USA: CO | Dolores River, 2 mi. S. Gateway, Mena Co. | 58 | A. mor. mor. | no data | 06-Sep-2001 | P.A. Opler, E.M. Buckner | 39.471 | -104.885 | 0 | 4 | h1169, 1170, 1171, 1172 |
| USA: CO | Peterson Creek, Saguache Co. | 59 | A. mej. pue. | no data | 11-Aug-1996 | R. E. Stanford | 38.306 | -105.984 | 0 | 2 | h1472 |
| USA: CO | Big Gypsum Valley, San Miguel Co. | 60 | A. mor. mor. | no data | 06-Sep-2001 | P.A. Opler, E.M. Buckner | 38.120 | -108.870 | 0 | 1 | h1169 |
| USA: NV | SE of Pahrump, Hwy 160 Mohave Desert, Clark Co. | 61 | A. mej. | no data | 30-Apr-2001 | Chuck & Cindy Harp | 36.026 | -115.691 | 0 | 1 | 1185 |
| USA: AZ | Reveg site, Cibola NWR, La Paz Co. | 62 | A. palmerii | no data | 19-Jun-1996 | S.M.N. | 33.307 | -114.704 | 0 | 1 | 1488 |
| USA: CA | Box Cyn., San Diego Co | 63 | C. wri. | no data | 12-Apr-2000 | J. Powell | 33.01 | -116.45 | 0 | 3 | 1401, 1402, 1403 |
| MEX: SO | Cananaea | 64 | A. mej. | no data | 22-Mar-2003 | P. Opler | 30.983 | -110.301 | 1 | 1 | 438 |
| MEX: SO | Cananaea | 64 | A. mej. | no data | 22-Mar-2003 | P. Opler | 30.983 | -110.301 | 1 | 1 | 439 |
| MEX: SO | Nacopuli Cyn., 5 mi N of San Carlos | 65 | A. mej. | no data | 23-Mar-2004 | P & E Opler | 28.388 | -111.312 | 0 | 3 | 1288, 1289, 1290 |
| MEX: SO | San Carlos, vic. Of Nacapuli Cyn. | 65 | A. mej. | no data | 26-Mar-2003 | J.Brock | 28.388 | -111.312 | 0 | 1 | 1287 |
| MEX: SO | Municipio Yecora | 66 | A. hep. | no data | 19-Mar-1998 | R. E. Stanford | 28.481 | -109.060 | 0 | 1 | 1479 |
| MEX: BS | Bahia Concepcion, Playa Santispac | 67 | C. wri., A. nr. hep. | no data | 07-Apr-2004 | E. Runquist | 26.870 | -111.918 | 0 | 3 | 1455, 1298, 1299 |
| MEX: SI | Chirimollos Hwy 40 | 68 | A. hep. | no data | 02-Dec-2003 | P & E Opler | 23.444 | -106.005 | 0 | 1 | 1292 |
| MEX: SI | Mazatlan | 69 | E. emesia | no data | 25-Nov-2002 | P. Opler | 23.321 | -106.402 | 0 | 2 | 1297, 1168 |

^a^A.: *Apodemia*; mor: *mormo*; lan.: *langei*; cyt.: *cythera*; tuo.: *tuolumnensis*; mej.: *mejicanus*; vir.: *virgulti*; nig.: *nigrescens*; pue.: *pueblo*; wri.: *wrighti*; hep.: *hepburni*; C.: *Calephelis*; E.: *Emesia*

*A. mor., A. mej., A. nr. mor., A. cyt. tuo.

Additional file 1: Table S2. Wing characters and descriptions.

| Character | State | | Description |
| --- | --- | --- | --- |
| FA | 0 |  | DF: Reduced orange scaling medially, obviously not reaching postmedian spot band |
|  | 1 |  | DF: Prominent orange scaling extending to or close to basal margin of postmedian spot band, or distal to no more than one spot |
|  | 2 | * | DF: Prominent orange scaling extending beyond more than one spot of postmedian spot band |
| FB | 0 |  | DF: Orange scaling very lightly present if at all anterior to discal cell |
|  | 1 | * | DF: Orange scaling extensively present anterior to discal cell |
| FC | 0 |  | DF: Orange scaling distant from anal margin: not proximal to 1st and 2nd spots from anal margin |
|  | 1 |  | DF: Orange scaling close to anal margin: proximal to 2nd but not 1st spot from anal margin |
|  | 2 | * | DF: Orange scaling very close to anal margin: proximal to 1st spot from anal margin |
| FD | 0 | * | DF: Basal spots reduced, substantially smaller than white postbasal spots |
|  | 1 |  | DF: Basal spots prominent, subequal to white postbasal spots |
| FE | 0 | * | DF: Anterior postbasal spot (in discal cell) white |
|  | 1 |  | DF: Anterior postbasal spot (in discal cell) invaded by orange scaling |
| FF | 0 | * | DF: Postmedian spots reduced, esp. spots 4 and 5 from the costal margin absent or nearly so |
|  | 1 |  | DF: Postmedian spots prominent |
| HG | 0 | * | DH: Orange scaling not present medially |
|  | 1 |  | DH: Orange scaling present medially |
| HH | 0 |  | DH: Orange scaling absent distal to postmedian spot band |
|  | 1 |  | DH: Orange scaling slightly present distal to postmedian spot band |
|  | 2 | * | DH: Orange scaling broadly present distal to postmedian spot band |
| HI | 0 | * | DH: White basal and postbasal spots reduced, esp. posterior basal spot greatly reduced if not absent (basal spots possibly obscured with orange scaling) |
|  | 1 |  | DH: All four white basal and postbasal spots prominent (basal spots possibly obscured with orange scaling) |
|  | 2 |  | DH: Postbasal spots obscured with orange scaling |
| HJ | 0 | * | DH: Postmedian spots reduced, several spots absent or nearly so |
|  | 1 |  | DH: Postmedian spots prominent |
| BK | 0 | * | DF: Marginal spots small, substantially smaller than postmedian spot band (if present) |
|  | 1 |  | DF: Marginal spots prominent, equal or subequal to postmedian spot band, esp. apical two |

In character names, "F" refers to a forewing character, "H" to a hindwing character, and "B" to a character on both pairs of wings. In the descriptions, "DF" refers to "dorsal forewing" and "DH" to "dorsal hindwing". Character states with asterisks refer to states illustrated in Figure 5.

Additional file 1: Table S3. Species descriptions: Descriptions and type localities of the 17 currently recognized subspecies within the *Apodemia mormo* species complex (Pelham 2008).

| Name | Description | Type locality^a^ | Latitude^b^ | Longitude^b^ |
| --- | --- | --- | --- | --- |
| *A. mormo mormo* | (Felder & Felder 1859) | Washoe Co., NV | 39.30 | -119.83 |
| *A. mormo cythera* | (Edwards 1873) | Independence, Inyo Co., CA | 36.599 | -118.059 |
| *A. mormo langei* | Comstock 1939 | Antioch, Contra Costa Co., CA | 38.0143 | -121.7933 |
| *A. mormo tuolumnensis* | Opler & Powell 1961 | Yosemite National Park, Tuolumne Co., CA | 37.739 | -119.569 |
| *A. mormo autumnalis* | Austin 1998 | Spring Mountains, Clark Co., NV | 35.83 | -115.43 |
| *A. mormo parva* | Austin 1998 | Diamond Mountains, Eureka Co., NV | 39.539 | -115.960 |
| *A. virgulti virgulti* | (Behr 1865) | La Tuna Canyon, Los Angeles Co., CA | 34.23 | -118.30 |
| *A. virgulti arenaria* | Emmel et al. 1998 | El Segundo sand dunes, Los Angeles Co., CA | 33.919 | -118.419 |
| *A. virgulti davenporti* | Emmel et al. 1998 | Walker Pass, Kern Co., CA | 35.659 | -118.029 |
| *A. virgulti dialeucoides* | Emmel et al. 1998 | Sugarloaf Mountain, San Bernardino Co., CA | 34.20 | -116.81 |
| *A. virgulti mojavelimbus* | Emmel et al. 1998 | Ord Mountains, San Bernardino Co., CA | 34.66 | -116.75 |
| *A. virgulti peninsularis* | Emmel et al. 1998 | Laguna Mountains, San Diego Co., CA | 32.88 | -116.44 |
| *A. virgulti nigrescens* | Emmel & Emmel 1998 | Colton, San Bernardino Co., CA | 34.069 | -117.310 |
| *A. virgulti pratti* | Emmel & Emmel 1998 | Holcomb Valley, San Bernardino Co., CA | 34.309 | -116.930 |
| *A. mejicanus mejicanus* | (Behr 1865) | Mazatlan, Sinaloa, Mexico | 23.22 | -106.40 |
| *A. mejicanus deserti* | Barnes & McDunnough 1918 | La Puerta Valley, San Diego Co., CA | 32.47 | -116.98 |
| *A. mejicanus pueblo* | Scott 1998 | Security, El Paso Co., CO | 38.764 | -104.735 |

^a^Type localities condensed from original description

^b^Coordinates estimated from description of type locality

LITERATURE CITED

Austin (1998) New subspecies of Lycaenidae (Lepidoptera) from Nevada and Arizona. In: Emmel TC (ed) Systematics of Western North American Butterflies. Mariposa Press, Gainesville, FL, pp 539-572

Barnes W, McDunnough (1918) Contributions to the Natural History of the Lepidoptera of North America 4:75

Edwards WH (1873) Descriptions of diurnal Lepidoptera found within the United States. Trans Am Entomol Soc 4:343-348

Emmel J, Emmel T (1998) Two new geographically restricted subspecies of *Apodemia mormo* (Lepidoptera: Riodinidae) from the vicinity of San Bernardino, California. In: Emmel TC (ed) Systematics of Western North American Butterflies. Mariposa Press, Gainesville, FL, pp. 795-800

Emmel J, Emmel T, Pratt G (1998) Five new subspecies of *Apodemia mormo* (Lepidoptera: Riodinidae) from southern California. In: Emmel TC (ed) Systematics of Western North American Butterflies. Mariposa Press, Gainesville, FL, pp. 801-810

Felder C, Felder R (1859) Lepidopterologische Fragmente. Wien Entomol Monatschr 3:271.

Pelham JP (2008) A catalogue of the butterflies of the United States and Canada with a complete bibliography of the descriptive and systematic literature. J Res Lepid 40:1–652

Scott, JA (1998) New western North American butterflies. Papilio (New Series) 11

Additional file 1: Table S4. Tajima’s D and Fu’s F statistics for Californian populations: Statistics for Pacific Northwest and eastern populations used in this study are presented in Proshek *et al.* 2013. N indicates sample size. No values were significant at *p* < 0.05.

| Locality # | N | Tajima’s D | Fu's F |
| --- | --- | --- | --- |
| 8 | 10 | -0.69098 | -0.59381 |
| 9 | 10 | -0.33956 | -0.34778 |
| 10 | 10 | 0 | 0 |
| 11 | 10 | -0.27922 | -1.58724 |
| 12 | 10 | 0.60162 | 0.48847 |
| 13 | 10 | 0.9258 | -0.27692 |
| 14 | 10 | 0.26356 | -1.51728 |
| 15 | 10 | -0.24329 | 0.39872 |
| 16 | 10 | -0.55215 | 0.22585 |
| 21 | 5 | -0.8165 | 0.09021 |
| 26 | 2 | 0 | 0 |
| 37 | 2 | 0 | 3.93183 |
| 38 | 10 | -0.61243 | -1.50959 |
| 41 | 11 | -0.38463 | -0.05362 |
| 42 | 10 | 1.74082 | 3.23684 |
| 64 | 2 | 0 | 0.69315 |

Additional file 1: Table S5. Pair-wise F_ST_ and heterozygosity values: Pair-wise F_ST_ values for all population pairs and observed and expected heterozygosities (before and after the slash, respectively) along the diagonal. Note, table can be increased in size to view entries.

Additional file 1: Table S6. Diagnostic wing characters: Diagnostic wing characters (see Additional file 1: Table S2 and Figure 5) scored for each of the 17 subspecies of the *Apodemia mormo* species complex recognized by Pelham (2008).

| Name | FA | FB | FC | FD | FE | FF | HG | HH | HI | HJ | BK |
| --- | --- | --- | --- | --- | --- | --- | --- | --- | --- | --- | --- |
| *A. mormo mormo** | 1 | 0 | 0 | 0 | 0 | 1 | 0 | 0 | 0 | 1 | 0 |
| *A. mormo cythera** | 2 | 1 | 2 | 0 | 0 | 1 | 0 | 2 | 1 | 1 | 1 |
| *A. mormo langei** | 1 | 1 | 2 | 0 | 1 | 1 | 1 | 0 | 2 | 1 | 0 |
| *A. mormo tuolumensis** | 2 | 1 | 2 | 0 | 0 | 0 | 0 | 2 | 0 | 0 | ? |
| *A. mormo autumnalis* | 1 | 1 | 1/2 | 0 | 0 | 1 | 0 | 0 | 1 | 0 | 1 |
| *A. mormo parva* | 1 | 1 | 1 | 0 | 0 | 1 | 0 | 0 | 1 | 1 | 0 |
| *A. virgulti virgulti** | 2 | 0 | 0 | 0 | 0 | 1 | 0 | 2 | 1 | 0 | 0 |
| *A. virgulti arenaria* | 2 | 0 | 1 | 0 | 0 | 0 | 0 | 2 | 0 | 1 | 0 |
| *A. virgulti davenporti* | 2 | 1 | 1 | 0 | 0 | 1 | 0 | 2 | 1 | ? | ? |
| *A. virgulti dialeucoides* | 0 | 0 | 0 | 1 | 0 | 1 | 0 | 0 | 1 | 1 | ? |
| *A. virgulti mojavelimbus* | 1 | 1 | 1 | 0 | 0 | 1 | 0 | 1 | 1 | 1 | 1 |
| *A. virgulti peninsularis* | 2 | 0 | 1 | 0 | 0 | 0 | 0 | 2 | 0 | 0 | 0 |
| *A. virgulti nigrescens** | ? | 0 | 0 | 0 | 0 | 0 | 0 | 1 | 0 | 0 | 0 |
| *A. virgulti pratti* | 2 | 1 | 0 | 1 | 0 | 1 | 0 | 2 | 1 | 1 | 1 |
| *A. mejicanus mejicanus** | 2 | 0/1 | 1/2 | 1 | 0 | 1 | 1 | 2 | 1 | 0/1 | 0 |
| *A. mejicanus deserti* | 2 | 1 | 1 | 1 | 0 | 1 | 0 | 0 | 1 | 1 | 1 |
| *A. mejicanus pueblo** | 2 | 0 | 0/1 | 1 | 0 | 1 | 1 | 2 | 1 | 0 | 0 |

* indicates a taxon for which genetic data was collected

? indicates a character that could not be scored for a taxon

Additional file 1: Table S7. Morphological dataset: Taxon names assigned to specimens of the *Apodemia mormo* species complex based on geographical information and 11 wing characters (see Additional file 1: Table S2 and Figure 5). Character states that could not be determined are displayed as “?”.

| ID No. | Assigned name | FA | FB | FC | FD | FE | FF | HG | HH | HI | HJ | BK |
| --- | --- | --- | --- | --- | --- | --- | --- | --- | --- | --- | --- | --- |
| 001 | *A. mormo nr. langei* | 1 | 1 | 1 | 0 | 1 | 1 | 0 | 0 | 2 | 1 | 1 |
| 002 | *A. mormo nr. langei* | 1 | 1 | 1 | 0 | 1 | 1 | 1 | 0 | 2 | 1 | 1 |
| 003 | *A. mormo nr. langei* | 1 | 1 | 1 | 0 | 0 | 0 | 0 | 0 | 2 | 0 | 1 |
| 004 | *A. mormo nr. langei* | 1 | 1 | 1 | 0 | 1 | 1 | 0 | 0 | 2 | 1 | 1 |
| 005 | *A. mormo nr. langei* | 1 | 1 | 1 | 0 | 1 | 1 | 0 | 0 | 2 | 1 | 1 |
| 006 | *A. mormo nr. langei* | 1 | 1 | 1 | 0 | 1 | 1 | 0 | 0 | 2 | 1 | 1 |
| 007 | *A. mormo nr. langei* | 1 | 1 | 1 | 0 | 1 | 1 | 0 | 1 | 2 | 1 | 1 |
| 008 | *A. mormo nr. langei* | 1 | 1 | 1 | 0 | 1 | 1 | 0 | 0 | 2 | 0 | 1 |
| 009 | *A. mormo nr. langei* | 1 | 1 | 1 | 0 | 1 | 1 | 0 | 0 | 2 | 1 | 1 |
| 010 | *A. mormo nr. langei* | 1 | 1 | 1 | 0 | 1 | 1 | 1 | 0 | 2 | 1 | 1 |
| 013 | *A. mormo* | 1 | 1 | 1 | 0 | 1 | 1 | 0 | 0 | 0 | 1 | 1 |
| 014 | *A. mormo* | 1 | 1 | 1 | 0 | 1 | 1 | 0 | 0 | 0 | 1 | 1 |
| 015 | *A. mormo* | 1 | 1 | 1 | 0 | 1 | 1 | 0 | 0 | 0 | 1 | 1 |
| 016 | *A. mormo* | 1 | 1 | 1 | 0 | 1 | 1 | 0 | 0 | 0 | 1 | 0 |
| 017 | *A. mormo* | 1 | 1 | 1 | 0 | 1 | 1 | 0 | 0 | 0 | 1 | 1 |
| 018 | *A. mormo* | 1 | 1 | 1 | 0 | 0 | 1 | 0 | 0 | 1 | 1 | 0 |
| 019 | *A. mormo* | 1 | 1 | 1 | 0 | 0 | 1 | 0 | 0 | 1 | 1 | 1 |
| 020 | *A. mormo* | 1 | 1 | 1 | 0 | 1 | 1 | 0 | 0 | 1 | 1 | 1 |
| 021 | *A. mormo* | 1 | 1 | 1 | 0 | 1 | 1 | 0 | 0 | 1 | 1 | 1 |
| 022 | *A. mormo* | 1 | 1 | 1 | 0 | 0 | 1 | 0 | 0 | 1 | 1 | 1 |
| 026 | *A. mormo* | 1 | 1 | 0 | 0 | 0 | 1 | 0 | 0 | 1 | 1 | 1 |
| 027 | *A. mormo* | 1 | 1 | 1 | 0 | 0 | 1 | 0 | 0 | 0 | 1 | 1 |
| 028 | *A. mormo* | 1 | 1 | 1 | 0 | 0 | 1 | 0 | 0 | 0 | 0 | 0 |
| 029 | *A. mormo* | 1 | 1 | 1 | 0 | 0 | 1 | 0 | 0 | 0 | 1 | 1 |
| 030 | *A. mormo* | 1 | 1 | 0 | 0 | 0 | 1 | 0 | 0 | 0 | 1 | 1 |
| 031 | *A. mormo* | 1 | 1 | 1 | 0 | 0 | 1 | 0 | 0 | 1 | 1 | 1 |
| 032 | *A. mormo* | 1 | 1 | 0 | 0 | 0 | 1 | 0 | 0 | 0 | 1 | 1 |
| 033 | *A. mormo* | 1 | 1 | 0 | 0 | 0 | 1 | 0 | 0 | 0 | 1 | 1 |
| 034 | *A. mormo* | 1 | 1 | 0 | 0 | 0 | 1 | 0 | 0 | 0 | 1 | 1 |
| 035 | *A. mormo* | 1 | 1 | 0 | 0 | 0 | 1 | 0 | 0 | 0 | 1 | 1 |
| 036 | *A. mormo* | 1 | 1 | 0 | 0 | 0 | 0 | 0 | 0 | 0 | 0 | 0 |
| 037 | *A. mormo* | 1 | 1 | 1 | 0 | 0 | 1 | 0 | 0 | 0 | 1 | 1 |
| 038 | *A. mormo* | 1 | 1 | 0 | 0 | 0 | 0 | 0 | 0 | 1 | 0 | 0 |
| 039 | *A. mormo* | 1 | 1 | 0 | 0 | 0 | 0 | 0 | 0 | 1 | 0 | 0 |
| 040 | *A. mormo* | 1 | 1 | 0 | 0 | 0 | 1 | 0 | 0 | 1 | 0 | 0 |
| 041 | *A. mormo* | 1 | 1 | 0 | 0 | 0 | 1 | 0 | 0 | 1 | 0 | 0 |
| 042 | *A. mormo* | 1 | 1 | 0 | 0 | 0 | 1 | 0 | 0 | 1 | 1 | 0 |
| 043 | *A. mormo* | 1 | 1 | 0 | 0 | 0 | 1 | 0 | 0 | 1 | 0 | 0 |
| 044 | *A. mormo* | 1 | 1 | 1 | 0 | 0 | 0 | 0 | 0 | 1 | 0 | 0 |
| 045 | *A. mormo* | 1 | 1 | 0 | 0 | 0 | 1 | 0 | 0 | 0 | 1 | 1 |
| 046 | *A. mormo* | 0 | 1 | 0 | 0 | 0 | 1 | 0 | 0 | 0 | 1 | 1 |
| 047 | *A. mormo* | 0 | 0 | 0 | 0 | 0 | 1 | 0 | 0 | 0 | 1 | 0 |
| 048 | *A. mormo* | 0 | 0 | 0 | 0 | 0 | 1 | 0 | 0 | 0 | 1 | 1 |
| 049 | *A. mormo* | 0 | 1 | 0 | 0 | 0 | 1 | 0 | 0 | 1 | 1 | 1 |
| 050 | *A. mormo* | 0 | 1 | 0 | 0 | 0 | 1 | 0 | 0 | 1 | 1 | 1 |
| 051 | *A. mormo* | 0 | 1 | 0 | 0 | 0 | 1 | 0 | 0 | 0 | 1 | 1 |
| 052 | *A. mormo* | 0 | 0 | 0 | 0 | 0 | 1 | 0 | 0 | 0 | 1 | 1 |
| 053 | *A. mormo* | 0 | 0 | 0 | 0 | 0 | 1 | 0 | 0 | 0 | 1 | 1 |
| 054 | *A. mormo* | 0 | 0 | 0 | 0 | 0 | 1 | 0 | 0 | 1 | 1 | 1 |
| 055 | *A. mormo* | 0 | 0 | 0 | 0 | 0 | 1 | 0 | 0 | 1 | 1 | 1 |
| 056 | *A. mormo* | 1 | 1 | 0 | 0 | 0 | 0 | 0 | 0 | 0 | 0 | 0 |
| 057 | *A. mormo* | 1 | 1 | 0 | 0 | 0 | 0 | 0 | 0 | 0 | 0 | 0 |
| 058 | *A. mormo* | 1 | 1 | 0 | 0 | 0 | 1 | 0 | 0 | 0 | 1 | 0 |
| 059 | *A. mormo* | 1 | 1 | 0 | 0 | 0 | 1 | 0 | 0 | 0 | 1 | 0 |
| 060 | *A. mormo* | 1 | 1 | 0 | 0 | 0 | 1 | 0 | 0 | 0 | 1 | 0 |
| 061 | *A. mormo* | 1 | 1 | 0 | 0 | 0 | 1 | 0 | 0 | 0 | 1 | 0 |
| 062 | *A. mormo* | 1 | 1 | 0 | 0 | 0 | 1 | 0 | 0 | 0 | 0 | 0 |
| 063 | *A. mormo* | 1 | 1 | 0 | 0 | 0 | 1 | 0 | 0 | 0 | 0 | 0 |
| 064 | *A. mormo* | 1 | 1 | 0 | 0 | 0 | 1 | 0 | 0 | 0 | 0 | 0 |
| 065 | *A. mormo* | 1 | 1 | 0 | 0 | 0 | 1 | 0 | 0 | 1 | 1 | 0 |
| 066 | *A. mormo* | 1 | 1 | 0 | 1 | 0 | 1 | 0 | 0 | 0 | 1 | 1 |
| 067 | *A. mormo* | 1 | 1 | 0 | 1 | 0 | 1 | 0 | 0 | 1 | 1 | 1 |
| 068 | *A. mormo* | 1 | 1 | 0 | 1 | 0 | 1 | 0 | 0 | 0 | 1 | 1 |
| 069 | *A. mormo* | 1 | 1 | 0 | 1 | 0 | 1 | 0 | 0 | 1 | 1 | 1 |
| 070 | *A. mormo* | 1 | 1 | 0 | 1 | 0 | 1 | 0 | 0 | 1 | 1 | 1 |
| 071 | *A. virgulti* | 2 | 1 | 0 | 0 | 0 | 1 | 0 | 2 | 0 | 0 | 1 |
| 072 | *A. virgulti* | 2 | 0 | 1 | 0 | 0 | 0 | 0 | 2 | 0 | 0 | 0 |
| 073 | *A. virgulti* | 2 | 0 | 1 | 0 | 0 | 0 | 0 | 2 | 0 | 0 | 0 |
| 074 | *A. virgulti* | 2 | 0 | 1 | 1 | 0 | 0 | 0 | 2 | 0 | 0 | 0 |
| 075 | *A. virgulti* | 2 | 1 | 1 | 1 | 0 | 1 | 0 | 2 | 0 | 0 | 0 |
| 076 | *A. virgulti* | 2 | 0 | 1 | 0 | 0 | 0 | 0 | 2 | 1 | 0 | 0 |
| 077 | *A. virgulti* | 2 | 1 | 0 | 0 | 0 | 1 | 0 | 2 | 1 | 0 | 0 |
| 078 | *A. virgulti* | 2 | 0 | 1 | 0 | 0 | 0 | 0 | 2 | 1 | 0 | 0 |
| 079 | *A. virgulti* | 2 | 0 | 1 | 0 | 0 | 0 | 0 | 2 | 1 | 0 | 0 |
| 080 | *A. virgulti* | 2 | 1 | 1 | 0 | 0 | 1 | 0 | 2 | 1 | 0 | 0 |
| 081 | *A. mormo* | 1 | 0 | 0 | 0 | 0 | 1 | 0 | 0 | 0 | 0 | 1 |
| 082 | *A. mormo* | 1 | 0 | 1 | 0 | 0 | 1 | 0 | 0 | 0 | 0 | 1 |
| 083 | *A. mormo* | 1 | 0 | 0 | 0 | 0 | 1 | 0 | 0 | 0 | 0 | 1 |
| 084 | *A. mormo* | 1 | 0 | 0 | 0 | 0 | 1 | 0 | 0 | 0 | 0 | 1 |
| 085 | *A. mormo* | 1 | 0 | 0 | 0 | 0 | 1 | 0 | 0 | 0 | 1 | 1 |
| 086 | *A. mormo* | 1 | 0 | 0 | 0 | 0 | 1 | 0 | 0 | 0 | 1 | 1 |
| 087 | *A. mormo* | 1 | 0 | 0 | 0 | 0 | 1 | 0 | 0 | 0 | 1 | 1 |
| 088 | *A. mormo* | 1 | 0 | 0 | 0 | 0 | 1 | 0 | 0 | 0 | 1 | 1 |
| 089 | *A. mormo* | 1 | 0 | 0 | 0 | 0 | 1 | 0 | 0 | 0 | 1 | 1 |
| 090 | *A. mormo* | 1 | 0 | 0 | 0 | 0 | 1 | 0 | 0 | 0 | 0 | 1 |
| 091 | *A. mormo langei* | 1 | 1 | 1 | 0 | 1 | 1 | 1 | 0 | 2 | 0 | 1 |
| 092 | *A. mormo langei* | 1 | 1 | 2 | 0 | 1 | 1 | 1 | 0 | 0 | 1 | 1 |
| 093 | *A. mormo langei* | 1 | 1 | 1 | 0 | 1 | 1 | 0 | 0 | 0 | 1 | 1 |
| 094 | *A. mormo langei* | 1 | 1 | 1 | 0 | 1 | 1 | 1 | 0 | 2 | 1 | 1 |
| 095 | *A. mormo langei* | 1 | 1 | 2 | 0 | 1 | 1 | 1 | 0 | 2 | 1 | 1 |
| 096 | *A. mormo langei* | 1 | 1 | 2 | 0 | 0 | 1 | 1 | 0 | 0 | 1 | 1 |
| 097 | *A. mormo langei* | ? | ? | ? | ? | ? | ? | ? | ? | ? | ? | ? |
| 098 | *A. mormo langei* | 1 | 1 | 1 | 0 | 1 | 1 | 0 | 0 | 0 | 0 | 1 |
| 099 | *A. mormo langei* | ? | ? | ? | ? | ? | ? | ? | ? | ? | ? | ? |
| 100 | *A. mormo langei* | 1 | 1 | 2 | 0 | 1 | 1 | 1 | 0 | 2 | 1 | 1 |
| 101 | *A. mormo* | 1 | 1 | 0 | 1 | 0 | 1 | 0 | 0 | 1 | 1 | 1 |
| 103 | *A. mormo* | 1 | 1 | 0 | 1 | 0 | 1 | 0 | 0 | 0 | 1 | 1 |
| 104 | *A. mormo* | 1 | 1 | 0 | 1 | 0 | 1 | 0 | 0 | 0 | 1 | 1 |
| 105 | *A. mormo* | 1 | 1 | 0 | 1 | 0 | 1 | 0 | 0 | 0 | 1 | 1 |
| 106 | *A. mormo* | 1 | 0 | 0 | 1 | 0 | 1 | 0 | 0 | 0 | 0 | 1 |
| 111 | *A. virgulti* | 2 | 0 | 0 | 0 | 0 | 0 | 0 | 2 | 0 | 0 | 1 |
| 112 | *A. virgulti* | 2 | 0 | 0 | 0 | 0 | 0 | 0 | 2 | 0 | 0 | 1 |
| 113 | *A. virgulti* | 2 | 0 | 0 | 0 | 0 | 0 | 0 | 2 | 0 | 0 | 1 |
| 114 | *A. virgulti* | 2 | 0 | 0 | 0 | 0 | 0 | 0 | 2 | 0 | 0 | 1 |
| 115 | *A. virgulti* | 2 | 0 | 0 | 0 | 0 | 0 | 0 | 2 | 0 | 0 | 1 |
| 116 | *A. virgulti* | 2 | 0 | 0 | 0 | 0 | 0 | 0 | 2 | 1 | 0 | 1 |
| 117 | *A. virgulti* | 2 | 0 | 0 | 0 | 0 | 0 | 0 | 2 | 0 | 0 | 1 |
| 118 | *A. virgulti* | 2 | 0 | 0 | 0 | 0 | 1 | 0 | 2 | 0 | 0 | 1 |
| 119 | *A. virgulti* | 2 | 0 | 0 | 0 | 0 | 1 | 0 | 2 | 0 | 0 | 1 |
| 120 | *A. virgulti* | ? | ? | ? | ? | ? | ? | ? | ? | ? | ? | ? |
| 121 | *A. virgulti* | 2 | 0 | 0 | 0 | 0 | 1 | 0 | 2 | 1 | 0 | 1 |
| 125 | *A. nr. mormo* | 1 | 0 | 0 | 1 | 0 | 1 | 0 | 0 | 1 | 1 | 1 |
| 126 | *A. nr. mormo* | 1 | 0 | 0 | 1 | 0 | 1 | 0 | 0 | 1 | 1 | 1 |
| 127 | *A. nr. mormo* | 1 | 1 | 0 | 1 | 0 | 1 | 0 | 1 | 1 | 1 | 1 |
| 128 | *A. nr. mormo* | 1 | 0 | 0 | 1 | 0 | 1 | 0 | 1 | 1 | 1 | 1 |
| 129 | *A. nr. mormo* | 1 | 0 | 0 | 1 | 0 | 1 | 0 | 0 | 1 | 1 | 1 |
| 130 | *A. nr. mormo* | ? | ? | ? | ? | ? | ? | ? | ? | ? | ? | ? |
| 131 | *A. nr. mormo* | 1 | 0 | 0 | 1 | 0 | 1 | 0 | 1 | 1 | 1 | 1 |
| 132 | *A. nr. mormo* | 1 | 0 | 0 | 1 | 0 | 1 | 0 | 0 | 1 | 1 | 1 |
| 133 | *A. nr. mormo* | 1 | 0 | 0 | 1 | 0 | 1 | 0 | 0 | 1 | 1 | 1 |
| 134 | *A. nr. mormo* | 1 | 0 | 0 | 1 | 0 | 1 | 0 | 0 | 1 | 1 | 1 |
| 412 | *A. cythera tuolumnensis* | 2 | 1 | 2 | 0 | 0 | 1 | 0 | 2 | 1 | 0 | 1 |
| 413 | *A. cythera* | 2 | 1 | 2 | 0 | 0 | 1 | 1 | 2 | 1 | 0 | 1 |
| 414 | *A. cythera tuolumnensis* | 2 | 1 | 2 | 0 | 0 | 1 | 0 | 2 | 0 | 0 | 1 |
| 415 | *A. cythera tuolumnensis* | 2 | 1 | 2 | 0 | 0 | 0 | 1 | 2 | 0 | 0 | 1 |
| 416 | *A. cythera tuolumnensis* | 2 | 1 | 2 | 0 | 0 | 1 | 0 | 2 | 1 | 0 | 1 |
| 418 | *A. cythera tuolumnensis* | 2 | 1 | 2 | 0 | 0 | 1 | 1 | 2 | 0 | 0 | 1 |
| 419 | *A. cythera* | 2 | 1 | 2 | 0 | 0 | 0 | 1 | 2 | 0 | 0 | 1 |
| 420 | *A. cythera* | 2 | 1 | 2 | 0 | 0 | 0 | 1 | 2 | 1 | 0 | 1 |
| 421 | *A. cythera tuolumnensis* | 2 | 1 | 2 | 0 | 0 | 1 | 1 | 2 | 0 | 0 | 1 |
| 422 | *A. cythera tuolumnensis* | 2 | 1 | 2 | 0 | 0 | 0 | 0 | 2 | 0 | 0 | 0 |
| 423 | *A. cythera* | 2 | 1 | 2 | 0 | 0 | 1 | 1 | 2 | 1 | 0 | 0 |
| 424 | *A. cythera* | 2 | 1 | 2 | 0 | 0 | 0 | 1 | 2 | 1 | 0 | 0 |
| 425 | *A. cythera tuolumnensis* | 2 | 1 | 2 | 0 | 0 | 0 | 0 | 2 | 0 | 0 | 0 |
| 426 | *A. cythera* | 2 | 1 | 2 | 0 | 0 | 0 | 1 | 2 | 0 | 0 | 0 |
| 427 | *A. cythera tuolumnensis* | 2 | 1 | 2 | 0 | 0 | 0 | 0 | 2 | 0 | 0 | 0 |
| 428 | *A. cythera* | 2 | 1 | 2 | 0 | 0 | 1 | 1 | 2 | 1 | 0 | 0 |
| 429 | *A. cythera tuolumnensis* | 2 | 1 | 2 | 0 | 0 | 0 | 1 | 2 | 0 | 0 | 0 |
| 430 | *A. cythera tuolumnensis* | 2 | 1 | 2 | 0 | 0 | 0 | 0 | 2 | 1 | 0 | 0 |
| 431A. | *A. mejicanus* | 1 | 1 | 1 | 0 | 0 | 1 | 0 | 0 | 1 | 1 | 1 |
| 431B. | *A. mejicanus* | 1 | 1 | 2 | 0 | 0 | 1 | 0 | 0 | 1 | 1 | 1 |
| 432A. | *A. mejicanus* | 1 | 1 | 2 | 0 | 0 | 1 | 0 | 0 | 1 | 1 | 1 |
| 432B. | *A. mejicanus* | 1 | 1 | 2 | 1 | 0 | 1 | 0 | 0 | 1 | 1 | 1 |
| 433A. | *A. mejicanus* | 1 | 1 | 2 | 1 | 0 | 1 | 0 | 0 | 1 | 1 | 1 |
| 433B. | *A. mejicanus* | 1 | 1 | 2 | 0 | 0 | 1 | 0 | 0 | 0 | 1 | 1 |
| 434 | *A. mejicanus* | 1 | 1 | 2 | 0 | 0 | 1 | 0 | 0 | 0 | 1 | 1 |
| 435A | *A. mejicanus* | 1 | 1 | 1 | 0 | 0 | 1 | 0 | 0 | 0 | 1 | 1 |
| 435B | *A. mejicanus* | 1 | 1 | 1 | 0 | 0 | 1 | 0 | 0 | ? | 1 | 1 |
| 435C | *A. mejicanus* | 1 | 1 | 1 | 0 | 0 | 1 | 0 | 0 | 1 | 1 | 1 |
| 436A | *A. mejicanus* | 1 | 1 | 2 | 0 | 0 | 1 | 0 | 0 | 0 | 1 | 1 |
| 436B | *A. mejicanus* | 1 | 1 | 1 | 0 | 0 | 1 | 0 | 0 | 0 | 1 | 1 |
| 437 | *A. mejicanus* | 1 | 0 | 1 | 0 | 0 | 1 | 0 | 0 | 0 | 1 | 1 |
| 438 | *A. mejicanus* | 1 | 1 | 1 | 0 | 0 | 1 | 0 | 2 | 0 | 1 | 1 |
| 439 | *A. mejicanus* | 1 | 1 | 1 | 1 | 0 | 1 | 0 | 2 | 0 | 1 | 1 |
| 0366 | *A. mejicanus pueblo* | 2 | 0 | 1 | 0 | 0 | 0 | 0 | 2 | 0 | 0 | 1 |
| 1142 | *A. mejicanus* | 1 | 1 | 1 | 1 | 0 | 1 | 0 | 0 | 1 | 1 | 1 |
| 1143 | *A. mejicanus* | 1 | 1 | 1 | 1 | 0 | 1 | 0 | 0 | 1 | 1 | 1 |
| 1148 | *A. virgulti* | 2 | 0 | 1 | 0 | 0 | 1 | 0 | 2 | 1 | 0 | 0 |
| 1149 | *A. virgulti* | 2 | 1 | 1 | 0 | 0 | 0 | 0 | 2 | 0 | 0 | 1 |
| 1150 | *A. virgulti* | 2 | 1 | 2 | 0 | 0 | 1 | 0 | 2 | 1 | 0 | 0 |
| 1151 | *A. virgulti* | 2 | 1 | 1 | 1 | 0 | 1 | 0 | 2 | 1 | 0 | 1 |
| 1152 | *A. virgulti* | 2 | 0 | 1 | 0 | 0 | 0 | 0 | 2 | 1 | 0 | 0 |
| 1153 | *A. virgulti* | 2 | 1 | 2 | 0 | 0 | 0 | 0 | 2 | 1 | 0 | 1 |
| 1154 | *A. virgulti* | 2 | 0 | 1 | 0 | 0 | 0 | 0 | 2 | 0 | 0 | 0 |
| 1155 | *A. mejicanus* | 2 | 1 | 1 | 1 | 0 | 1 | 0 | 1 | 1 | 1 | 1 |
| 1156 | *A. nr. mormo* | 1 | 1 | 2 | 0 | 0 | 1 | 0 | 0 | 1 | 1 | 1 |
| 1157 | *A. virgulti* | 1 | 1 | 2 | 0 | 0 | 1 | 0 | 1 | 1 | 0 | 1 |
| 1158 | *A. mejicanus* | 2 | 1 | 2 | 0 | 0 | 1 | 0 | 1 | 0 | 0 | 1 |
| 1159 | *A. nr. mormo* | 2 | 1 | 2 | 0 | 0 | 1 | 0 | 0 | 1 | 1 | 0 |
| 1160 | *A. cythera tuolumnensis* | 2 | 1 | 2 | 0 | 0 | 1 | 0 | 2 | 1 | 0 | 0 |
| 1161 | *A. cythera* | 2 | 1 | 2 | 0 | 0 | 1 | 0 | 2 | 0 | 0 | 0 |
| 1162 | *A. cythera tuolumnensis* | 2 | 1 | 2 | 0 | 0 | 0 | 0 | 2 | 1 | 0 | 0 |
| 1163 | *A. cythera tuolumnensis* | 2 | 1 | 2 | 0 | 0 | 0 | 0 | 2 | 1 | 0 | 0 |
| 1164 | *A. cythera* | 2 | 1 | 1 | 0 | 0 | 1 | 0 | 2 | 1 | 1 | 1 |
| 1165 | *A. cythera* | 2 | 1 | 2 | 0 | 0 | 1 | 0 | 2 | 1 | 0 | 0 |
| 1166 | *A. cythera* | 2 | 1 | 2 | 0 | 0 | 1 | 1 | 2 | 0 | 1 | 1 |
| 1167 | *A. virgulti* | 2 | 0 | 0 | 0 | 0 | 0 | 0 | 2 | 0 | 0 | 1 |
| 1169 | *A. mormo* | 1 | 0 | 1 | 0 | 0 | 1 | 0 | 0 | 1 | 1 | 0 |
| 1170 | *A. mormo* | 1 | 0 | 1 | 0 | 0 | 1 | 0 | 0 | 0 | 1 | 0 |
| 1171 | *A. mormo* | 1 | 0 | 0 | 0 | 0 | 1 | 0 | 0 | 0 | 1 | 0 |
| 1172 | *A. mormo* | 1 | 1 | 1 | 0 | 0 | 1 | 0 | 0 | 0 | 0 | 0 |
| 1173 | *A. mormo* | ? | ? | ? | ? | ? | ? | ? | ? | ? | ? | ? |
| 1174 | *A. mejicanus* | 2 | 1 | 2 | 0 | 0 | 1 | 0 | 1 | 0 | 0 | 0 |
| 1175 | *A. nr. mormo* | 2 | 0 | 2 | 0 | 0 | 1 | 0 | 0 | 1 | 1 | 1 |
| 1176 | *A. nr. mormo* | 2 | 1 | 1 | 0 | 0 | 1 | 0 | 0 | 0 | 1 | 1 |
| 1177 | *A. cythera tuolumnensis* | 2 | 1 | 2 | 0 | 0 | 1 | 0 | 2 | 1 | 1 | 1 |
| 1178 | *A. nr. mormo* | 2 | 1 | 2 | 0 | 0 | 1 | 0 | 0 | 1 | 1 | 1 |
| 1179 | *A. mormo* | 1 | 0 | 1 | 0 | 0 | 1 | 0 | 0 | 1 | 1 | 0 |
| 1180 | *A. virgulti nigrescens* | 2 | 0 | 0 | 0 | 0 | 0 | 0 | 2 | 1 | 0 | 0 |
| 1181 | *A. virgulti nigrescens* | 2 | 0 | 0 | 0 | 0 | 0 | 0 | 2 | 0 | 0 | 0 |
| 1182 | *A. virgulti nigrescens* | 2 | 0 | 0 | 0 | 0 | 0 | 0 | 2 | 1 | 0 | 0 |
| 1183 | *A. virgulti nigrescens* | 2 | 0 | 0 | 0 | 0 | 0 | 0 | 2 | 0 | 0 | 0 |
| 1184 | *A. virgulti nigrescens* | 2 | 0 | 0 | 0 | 0 | 0 | 0 | 2 | 1 | 0 | 0 |
| 1185 | *A. mejicanus* | 1 | 1 | 2 | 1 | 0 | 1 | 0 | 1 | 1 | 1 | 1 |
| 1186 | *A. cythera* | 2 | 1 | 2 | 0 | 0 | 0 | 1 | 2 | 0 | 0 | 0 |
| 1187 | *A. cythera* | 2 | 1 | 2 | 0 | 0 | 0 | 1 | 2 | 1 | 0 | 0 |
| 1188 | *A. cythera* | 2 | 1 | 2 | 0 | 0 | 1 | 0 | 2 | 1 | 0 | 0 |
| 1189 | *A. cythera* | 2 | 1 | 2 | 0 | 0 | 1 | 0 | 2 | 1 | 0 | 0 |
| 1190 | *A. cythera* | 2 | 1 | 2 | 0 | 0 | 0 | 1 | 2 | 1 | 0 | 0 |
| 1287 | *A. mejicanus* | 2 | 0 | 2 | 1 | 0 | 1 | 1 | 2 | 1 | 1 | 1 |
| 1288 | *A. mejicanus* | 2 | 0 | 2 | 1 | 0 | 1 | 1 | 2 | 1 | 1 | 1 |
| 1289 | *A. mejicanus* | 2 | 0 | 0 | 1 | 0 | 1 | 0 | 1 | 1 | 1 | 1 |
| 1290 | *A. mejicanus* | 2 | 0 | 1 | 1 | 0 | 1 | 1 | 2 | 1 | 1 | 1 |
| 1448 | *A. cythera* | 2 | 1 | 2 | 0 | 0 | 0 | 1 | 2 | 0 | 0 | 0 |
| 1471 | *A. mejicanus* | 2 | 1 | 1 | 0 | 0 | 1 | 1 | 1 | 0 | 1 | 1 |
| 1472 | *A. mejicanus pueblo* | 2 | 0 | 0 | 1 | 0 | 1 | 0 | 2 | 1 | 0 | 0 |
| 1473 | *A. mejicanus pueblo* | 2 | 0 | 0 | 1 | 0 | 0 | 1 | 2 | 0 | 0 | 0 |
| 1474 | *A. virgulti* | 2 | 0 | 1 | 0 | 0 | 0 | 0 | 2 | 0 | 0 | 0 |
| 1475 | *A. virgulti* | 2 | 0 | 1 | 0 | 0 | 0 | 0 | 2 | 1 | 0 | 0 |
| 1476 | *A. cythera* | 2 | 1 | 1 | 0 | 0 | 0 | 1 | 2 | 1 | 0 | 1 |
| 1477 | *A. virgulti* | 2 | 0 | 1 | 0 | 0 | 0 | 0 | 2 | 1 | 0 | 0 |
| 1478 | *A. cythera* | 2 | 1 | 2 | 0 | 0 | 1 | 0 | 2 | 0 | 0 | 0 |

Additional file 1: Table S8. Summary of the six major sources of specimens.

|  | Collectors | N | Region | Dates | Preservation^a^ | Extraction^b^ | Locations^c^ | Vouchers^d^ |
| --- | --- | --- | --- | --- | --- | --- | --- | --- |
| 1 | B. Proshek *et al*. | 273 | BC, SK, MT, ND, SD, WY, ID, OR, WA | Aug 2007 - Sep 2008 | two legs in 99% ethanol | DNeasy | 01, 03-07, 43-54 | UASM, GNP |
| 2 | F. Sperling, J. Powell *et al*. | 121 | California | Sep 1995 - Jun 1998 | Live frozen | Phenol-chloroform | 08-16. 38, 41-42 | UASM, EME |
| 3 | P. Opler, K. Davenport *et al*. | 82 | CA, AZ, NM, CO, Mexico | Jul 1992 - Sep 2008 | Dried | CCDB | 18, 20, 22-24, 27, 29-36, 39-40, 55-62, 64-69 | CSU |
| 4 | J. Powell | 12 | California | Apr 2000 | Dried | DNeasy | 26, 28, 37, 63 | UASM |
| 5 | K. Davenport | 17 | California | Aug 1989 - Nov 2002 | Dried | DNeasy | 17, 19, 21, 25 | UASM |
| 6 | L. Crawford, S. Desjardins | 42 | BC, WA | Aug 2008 | wing clips in 99% ethanol | DNeasy | 01 | N/A |

^a^Initial preservation method

^b^DNeasy: DNeasy Tissue Extraction Kit (Qiagen, Valencia, CA); CCDB: extractions processed according to the methods of the Canadian Centre for DNA Barcoding (Guelph, ON) (Hajibabaei et al. 2005, [www.dnabarcoding.ca](http://www.dnabarcoding.ca/))

^c^Location numbers refer to those in Figure 2 and Additional file 1: Table S1

^d^UASM: University of Alberta, Strickland Museum of Entomology; EME: University of California, Berkeley, Essig Museum of Entomology; CSU: Colorado State University, C.P. Gillette Museum; GNP: Grasslands National Park

LITERATURE CITED

Crawford LA, Desjardins S, Keyghobadi N: Fine-scale genetic structure of an endangered population of the Mormon metalmark butterfly (Apodemia mormo) revealed using AFLPs. Conserv Genet 2011, 12:991–1001.

Additional file 1: Table S9. GenBank accession numbers.

| DNA # | Accession |
| --- | --- |
| 001 | KP262883 |
| h002 | KP262884 |
| h003 | KP262885 |
| 004 | KP262886 |
| 006 | KP262887 |
| 007 | KP262888 |
| h013 | KP262889 |
| h017 | KP262890 |
| 018 | KP262891 |
| 019 | KP262892 |
| 020 | KP262893 |
| 022 | KP262894 |
| h026 | KP262895 |
| 027 | KP262896 |
| 028 | KP262897 |
| 029 | KP262898 |
| h031 | KP262899 |
| 032 | KP262900 |
| 033 | KP262901 |
| 035 | KP262902 |
| h036 | KP262903 |
| h038 | KP262904 |
| 039 | KP262905 |
| h040 | KP262906 |
| h046 | KP262907 |
| 053 | KP262908 |
| 054 | KP262909 |
| 055 | KP262910 |
| h056 | KP262911 |
| h058 | KP262912 |
| h059 | KP262913 |
| 060 | KP262914 |
| h061 | KP262915 |
| h066 | KP262916 |
| h071 | KP262917 |
| 072 | KP262918 |
| h073 | KP262919 |
| 079 | KP262920 |
| h081 | KP262921 |
| h082 | KP262922 |
| h083 | KP262923 |
| 085 | KP262924 |
| 091 | KP262925 |
| h092 | KP262926 |
| 093 | KP262927 |
| h094 | KP262928 |
| h096 | KP262929 |
| 111 | KP262930 |
| 112 | KP262931 |
| 113 | KP262932 |
| h114 | KP262933 |
| 115 | KP262934 |
| 116 | KP262935 |
| 120 | KP262936 |
| 121 | KP262937 |
| 125 | KP262938 |
| 126 | KP262939 |
| 127 | KP262940 |
| h128 | KP262941 |
| 130 | KP262942 |
| 131 | KP262943 |
| 132 | KP262944 |
| h135 | KP262957 |
| 136 | KP262958 |
| h137 | KP262959 |
| h139 | KP262960 |
| 140 | KP262961 |
| h156 | KP262962 |
| h157 | KP262963 |
| h166 | KP262964 |
| 171 | KP262965 |
| h178 | KP262969 |
| h180 | KP262966 |
| h196 | KP262970 |
| h197 | KP262971 |
| 198 | KP262972 |
| 199 | KP262973 |
| h202 | KP262974 |
| 206 | KP262975 |
| h207 | KP262976 |
| 208 | KP262977 |
| 212 | KP262978 |
| 213 | KP262979 |
| h214 | KP262980 |
| 216 | KP262981 |
| 217 | KP262982 |
| 218 | KP262983 |
| h219 | KP262984 |
| 220 | KP262985 |
| h223 | KP262986 |
| h225 | KP262987 |
| h226 | KP262988 |
| 228 | KP262989 |
| 232 | KP262990 |
| h237 | KP262991 |
| 240 | KP262992 |
| h254 | KP262993 |
| 255 | KP262994 |
| 256 | KP262995 |
| 258 | KP262996 |
| h259 | KP262997 |
| 260 | KP262998 |
| 261 | KP262999 |
| h265 | KP263000 |
| 274 | KP263001 |
| 278 | KP263002 |
| h281 | KP263003 |
| 283 | KP263004 |
| 285 | KP263005 |
| 286 | KP263006 |
| h287 | KP263007 |
| h295 | KP263008 |
| h297 | KP263009 |
| 298 | KP263010 |
| 300 | KP263011 |
| h307 | KP263012 |
| 309 | KP263013 |
| h311 | KP263014 |
| h318 | KP263015 |
| 321 | KP263016 |
| h323 | KP263017 |
| 326 | KP263018 |
| h329 | KP263019 |
| h330 | KP263020 |
| h332 | KP263021 |
| 334 | KP263022 |
| h335 | KP263023 |
| h341 | KP263024 |
| 343 | KP263025 |
| 344 | KP263026 |
| h345 | KP263027 |
| 346 | KP263028 |
| h350 | KP263029 |
| 352 | KP263030 |
| h356 | KP263031 |
| 358 | KP263032 |
| h371 | KP263033 |
| 400 | KP262967 |
| 405 | KP262968 |
| h412c | KP262945 |
| h412ct | KP262946 |
| 414 | KP262947 |
| 415 | KP262948 |
| 419 | KP262949 |
| 421 | KP262950 |
| 422 | KP262951 |
| 425 | KP262952 |
| 429 | KP262953 |
| h431A | KP262954 |
| h431B | KP262955 |
| 435B | KP262956 |
| 435C | KP263035 |
| 438 | KP263036 |
| 439 | KP263037 |
| SHK02 | KP263034 |
| 1142 | GU685512 |
| 1143 | GU685513 |
| 1148 | GU685510 |
| 1149 | GU685511 |
| 1150 | GU685504 |
| 1151 | GU685505 |
| 1152 | GU685506 |
| 1153 | GU685507 |
| 1154 | GU685500 |
| 1155 | GU685501 |
| 1156 | GU685502 |
| 1157 | GU685503 |
| 1158 | GU685496 |
| 1159 | GU685497 |
| 1160 | GU685498 |
| 1161 | GU685499 |
| 1162 | GU685492 |
| 1163 | GU685493 |
| 1164 | GU685494 |
| 1165 | GU685495 |
| 1166 | GU685488 |
| 1167 | GU685489 |
| 1168 | GU685490 |
| 1169 | GU685491 |
| 1170 | GU685484 |
| 1171 | GU685485 |
| 1172 | GU685486 |
| 1173 | GU685487 |
| 1174 | GU685480 |
| 1175 | GU685481 |
| 1176 | GU685482 |
| 1177 | GU685483 |
| 1178 | GU685476 |
| 1179 | GU685477 |
| 1180 | GU685478 |
| 1181 | GU685479 |
| 1182 | GU685472 |
| 1183 | GU685473 |
| 1184 | GU685474 |
| 1185 | GU685475 |
| 1186 | GU685468 |
| 1187 | GU685469 |
| 1188 | GU685470 |
| 1189 | GU685471 |
| 1190 | GU685464 |
| 1287 | GU685372 |
| 1288 | GU685373 |
| 1289 | GU685374 |
| 1290 | GU685375 |
| 1292 | HM372885 |
| 1293 | GU685369 |
| 1294 | GU685370 |
| 1295 | GU685371 |
| 1296 | GU685365 |
| 1297 | GU685366 |
| 1298 | GU685367 |
| 1299 | GU685368 |
| 1448 | GU685230 |
| 1470 | HM417822 |
| 1471 | GU685214 |
| 1472 | HM914974 |
| 1473 | GU685215 |
| 1474 | GU685216 |
| 1475 | GU685213 |
| 1476 | GU685211 |
| 1477 | GU685212 |
| 1478 | GU685208 |
| 1479 | HM372889 |
| 1487 | GU685201 |
| 1488 | HM372890 |
